# Development of a sensitive, quantitative assay with broad subtype specificity for detection of total HIV-1 nucleic acids in plasma and PBMC

**DOI:** 10.1038/s41598-021-03016-1

**Published:** 2022-01-28

**Authors:** C. N. Kibirige, M. Manak, D. King, B. Abel, H. Hack, D. Wooding, Y. Liu, N. Fernandez, J. Dalel, Steve Kaye, N. Imami, L. Jagodzinski, J. Gilmour

**Affiliations:** 1 IAVI, Human Immunology Laboratory, Imperial College London, Chelsea and Westminster NHS Foundation Trust, 369 Fulham Road, London, SW10 9NH UK; 2 Henry M. Jackson Foundation for the Advancement of Military Medicine, Bethesda, MD USA; 3 U.S. Military HIV Research Program, Walter Reed Army Institute of Research, 503 Robert Grant Ave., Silver Spring, MD 20910 USA; 4 Diagnostics and Countermeasures Branch, Walter Reed Army Institute of Research, 503 Robert Grant Ave, Silver Spring, MD 20910 USA; 5 Molecular Diagnostics Unit, Imperial College London, Jefferiss Trust Laboratory, St. Mary’s Campus, Norfolk Place, London, W2 1PG UK; 6 Centre for Immunology and Vaccinology, Imperial College London, Chelsea and Westminster NHS Foundation Trust, 369 Fulham Road, London, SW10 9NH UK; 7 Present Address: Turesol Consulting, 314 S. Henderson Road, King of Prussia, PA 19406 USA

**Keywords:** Biological techniques, Molecular biology, Biomarkers, Diseases, Health care, Medical research, Molecular medicine, Pathogenesis

## Abstract

An LTR-based quantitative PCR (qPCR) assay was modified and optimized for the quantification of total HIV-1 nucleic acids in plasma and PBMC. TaqMan qPCR primers and probes were designed against the NCBI/LANL HIV-1 compendium database by analyzing sequences used in assays for sensitive cross-clade detection of HIV-1 as reported in the literature and elucidating regions of improved cross-subtype specificity. Inosine and mixed nucleotide bases were included at polymorphic sites. Real-time RT-qPCR and qPCR were performed on plasma viral RNA and cellular lysates. A step-up amplification approach to allow binding of primers across polymorphic regions showed improved sensitivity compared to universal cycling. Unlike a lead competing laboratory-developed assay, all major HIV-1 subtypes, and a wide range of recombinants from a 127-member diversity panel were detected and accurately quantified in spiked plasmas. Semi-nested PCR increased detection sensitivity even further. The assay was able to detect down to 88 copies/mL of HIV-1 in plasma with 95% efficiency or the equivalent of a single infected cell. The PCR assay will be valuable in studies that monitor very low viral levels including residual or break through HIV-1 in patients receiving antiretroviral therapy, in HIV-1 cure, and in other research studies.

## Introduction

In order to effectively monitor HIV-1 combination anti-retroviral therapy (cART) and support HIV-1 cure studies internationally, there is a need for relatively simple low-cost sensitive assays that can reliably quantify low levels of HIV-1, across different viral subtypes^1–3^. HIV suppression, following initiation of cART, to levels that are undetectable by commercial viral load assays (i.e., below 20–50 copies/ml in plasma), is significantly associated with longevity and improved quality of life. Moreover, since successfully treated people do not transmit the virus, early and sustained viral suppression is an important public health tool in the prevention of the spread of HIV. Current WHO guidelines thus strongly recommend initiation of cART as soon as possible after HIV-1 diagnosis^4^.

Interestingly, the use of highly sensitive laboratory developed assays (LDAs), such as the Single-Copy Assay (SCA) which detects down to 1 copy of HIV-1 RNA per ml of plasma, has shown that discontinuation of antiretroviral treatment by HIV-infected patients with plasma RNA levels above 10–15 copies/ml results in viremia rebound earlier and more often than patients who have lower or undetectable RNA^5,6^. These results indicate that candidates for viral control or cure studies may have better success if viral loads are driven below 15 copies/ml before treatment cessation.

HIV-1 DNA integrates into the human chromosome and persists in an inactive state even in the presence of ongoing antiretroviral therapy. This means that reduction of plasma viral load to low levels, even below 10–15 copies/ml as indicated by the highly efficient SCA assay, does not lead to viral eradication. The integrated viral DNA serves as a long-term reservoir for viral reinfection and can be reactivated when therapy is discontinued or when drug-resistant isolates emerge^6–8^. Despite dramatic decreases in plasma HIV-1 RNA levels following treatment with cART, levels of integrated viral DNA in virally suppressed subjects persist at similar levels to those of untreated subjects^9^. HIV-1 viral reservoir sites include CD4 memory T cells, lymph nodes, the gut and other tissues^8^. To measure the levels of replication-competent integrated DNA within these reservoir sites, various assays have been developed. The quantitative virus outgrowth assay (QVOA) is considered the gold-standard assay for this type of assessment, but is complicated, expensive and time intensive, and despite recent improvements, is not practical for routine viral monitoring^10^.

Alternative assays have been developed to quantify cell-associated viral nucleic acids for measurement of residual quiescent viral genomes^11^. Indeed, several studies have shown that viral nucleic acids can be readily detected in whole blood and peripheral blood mononuclear cells (PBMCs) of HIV-1 infected individuals even when plasma viral load is very low or undetectable^12–14^. The level of residual HIV-1 DNA in the PBMCs of treated subjects ranges from 100 to 10,000 copies of HIV-1 DNA per million cells and remains sufficiently high as to allow for reliable detection of quiescent infection, with only those who have initiated therapy during Fiebig disease stage I or II at lower levels^12,14^. These alternative cell-based PCR assays have been shown to correlate well with the QVOA and thus the latent viral reservoir^6,15^. These simplified assays are used as a proxy for viral reservoir estimation and provide a more efficient tool for large-scale patient screening and research because they can be tailored to the available specimens and can be run at lower cost (~ < $4 per test) compared to FDA-approved plasma viral load assays ($50–100+ per test)^16,17^.

Another lingering issue in global HIV-1 treatment and monitoring is that many of the original LDAs for HIV-1 quantification used in clinical research were developed against HIV-1 subtype B, the predominant subtype circulating in Europe and America. This subtype only accounts for 10% of the infections worldwide^18^. Rapid spread of HIV in Africa and Asia and increased international travel have led to a steady increase in non-B HIV-1 in the western hemisphere^19^. Current HIV-1 isolates have been classified into three major groups (M, N and O) and further subdivided into subtypes or clades, various circulating recombinant forms (CRFs) and unique recombinant forms (URFs) spanning up to 40% sequence divergence^20^. A major challenge in the design and implementation of LDAs for HIV-1, therefore, is the large worldwide sequence diversity of the virus and the lack of ability of current LDAs to cover the broad range of HIV-1 subtypes in various target populations. Moreover, the extensive variability in sample processing and amplification procedures among the various LDAs, makes it difficult to compare subtype sensitivity and specificity between different test formats and laboratories. Several programs, such as the HIV Reservoir Assay Validation and Evaluation Network (RAVEN) project (www.cavd.org), and the NIH/NIAID/DIAIDS External Quality Assurance Program Oversight Laboratory (EQAPOL) (https://eqapol.dhvi.duke.edu/) have emerged to provide standardized platforms for assay validation and to provide guidance for the minimum information needed for publication of quantitative real-time PCR experiments (MIQE)^21^. These QC programs and reagents help ensure assay claims and allow comparison of performance across different assays and laboratories.

Here, we describe a sensitive polymerase chain reaction (PCR)-based assay which can be used in various formats to detect residual HIV-1 nucleic acids in the plasma and PBMCs of HIV-1 infected individuals undergoing cART. In the traditional non-nested liquid format, the assay can detect 88 copies of HIV-1 RNA per ml with 95% efficiency. A semi-nested liquid format allows for greater sensitivity. In the alternative cellular format, the assay can detect down to 3 input copies of nucleic acid or the equivalent of a single infected cell at the 95% confidence level. Compared to previously reported LDAs, our assay provides improved efficiency, a very broad dynamic range and much improved cross-subtype specificity^9,22–24^. We have evaluated this assay according to MIQE guidelines using an extensive diversity panel of HIV-1 strains provided by EQAPOL and the United States Military HIV Research Program (US MHRP) via the AIDS Reagent Program. It has comparable sensitivity to current commercial PCR-based HIV-1 assays used for plasma viral load testing and for sensitive detection of cell-associated HIV-1 levels in PBMCs but is considerably cheaper to perform which greatly increases its utility in resource-limited settings. Please note that this technology is the subject of a priority patent application. Any parties wishing to commercialize these assays or variations thereof, please contact Imperial College London via enterprise@imperial.ac.uk, citing reference number 10579.

## Results

### Assay design

Oligonucleotide sequence names are denoted by the first nucleotide of their HXB2 reference sequence base position and an “F” for forward primer, “P” for probe or “R” for reverse primer. The main oligonucleotide sets studied during this project are also denoted by the first author and their year of publication e.g., Brussel 2005.

### Previously reported laboratory developed assay

Initial screening of published nucleotides found that the 496F/546P/633R primer–probe set (Brussel 2005), with an optional Friedrich 2010 internal reverse primer 622R produced the best and most consistent results but showed limited specificity for non-subtype B isolates on a sub-panel of 20 EQAPOL isolates that were difficult to detect and accurately quantify (Table 1). Table 1 shows data on the 8 most problematic strains.

**Table 1:** Effect of nested PCR, step-up cycling, and oligonucleotide sequence on cross-subtype specificity and accuracy of HIV-1 RT-qPCR LDAs.

Accession	Subtype	Country of origin	Year	[Expected] Copies/ml	[Observed] Copies/mL–Laboratory developed assays				
					Brussel/Freidrich assay				Revised assay
				Roche Cobas	Non-nested universal	Nested universal	Non-nested step-up	Nested step-up	Semi-nested step-up
JX140676	G	Cameroon	2010	**1.53e + 05**	^◦^1.65e + 01	^◦^1.80e + 01	0.00e + 00	^◦^3.48e + 02	**1.17e + 05**
KC596065	CRF01_AE	China	2011	**6.58e + 04**	0.00e + 00	0.00e + 00	0.00e + 00	0.00e + 00	0.00e + 00
KF859742	O	Germany	2012	**5.42e + 04**	0.00e + 00	0.00e + 00	0.00e + 00	0.00e + 00	^◦^4.45e + 03
KF859745	A1	Uganda	2010	**1.43e + 04**	0.00e + 00	^◦^1.53e + 03	^◦^4.41e + 02	^◦^5.72e + 02	**5.98e + 04**
KF716467	C	Zambia	2011	**5.25e + 04**	^◦^2.15e + 03	^◦^1.65e + 03	^◦^3.46e + 02	^◦^8.70e + 02	**9.81e + 04**
KF716488	A1, G, CRF01_AE	Uganda	2010	**5.04e + 04**	0.00e + 00	0.00e + 00	0.00e + 00	0.00e + 00	**2.89e + 05**
KP109492	URF_A1D	Uganda	2009	**2.81e + 04**	0.00e + 00	^◦^6.54e + 02	^◦^5.55e + 01	^◦^6.07e + 02	**6.11e + 04**
KU749414	A1, G	Pakistan	2014	**1.21e + 05**	^◦^1.86e + 03	^◦^1.24e + 03	^◦^6.70e + 02	^◦^1.12e + 03	**4.14e + 05**
Assay efficiency%					99.98	93.73	94.81	89.67	> 95.00
~ LOD/LOQ95 (based on input copies of RNA via standard curve, not copies/ml)					30/3e3	1/30	1/12.5	1/3	1/3

NB: This table includes a selection of 8 HIV-1 strains that were difficult to quantify using the Brussel/Freidrich assay—496F/546P/622R1/633R2, compared to the performance of the revised 525F/574P/599R assay. 10 ng/ml tRNA was used in the mastermix, in all these experiments.

^◦^Samples that were under-quantified by the LDAs; Bold type = Samples that were correctly quantified by the assays.

Further optimization, including adjustment of oligonucleotide concentration, utilization of nested PCR, step-up cycling and addition of tRNA to the master-mix, resulted in improved assay performance with a standard curve of > 90% efficiency, a linear range over 5 logs and a LLOD/LLOQ95 of 3 input copies of HIV RNA, but did not enhance the detection of non-detectable viral strains (Table 1).

Subsequent sequence analysis of these oligonucleotide sets against the HIV-1 LANL compendium database, showed significant mismatch issues (Supplementary Table S2) and indicated the need to revise the assay oligonucleotides.

### Revised laboratory developed assay

A revised set of oligonucleotides was designed using AlleleID 7.0 software and the IDT Oligoanalyzer tool as detailed in the methods. The revised sequences can be found in Table 2.

**Table 2:** Oligonucleotides designed and tested for various formats of the HIV-1 assay.

Oligo name	HXB2^a^ nt position	Assay component, assay format and target (see also Supplementary Table S4)	Sequence
525F	LTR 525 → 543	Forward primer (non-nested RNA or DNA)	5′-TCAATAAAGCTTGCCTTGA-3′
λ525F	LTR 525 → 543	Forward primer round 1 (semi-nested RNA or DNA)	5′-ATGCCACGTAAGCGAAACTTCAATAAAGCTTGCCTTGA-3′
λT	n/a	Forward primer round 2 (semi-nested RNA or DNA)	5′-ATGCCACGTAAGCGAAACT-3′
574P	LTR 574 ← 552	Probe (total RNA or DNA)	5′-FAM/ACAGAYGGGCACACAC**N**ACT/MGBNFQ-3′
599R	LTR 599 ← 582	Reverse primer (total RNA or DNA)	5′-AGGGATCTCTAG**N**TACCA-3′

^a^Genbank Accession ID K03455. Inosine and mixed bases shown in enlarged bold font. AlleleID 7.0 and IDT Oligoanalyzer software utilized.

### Instrument, and probe–chemistry optimization

**Figure 1:**
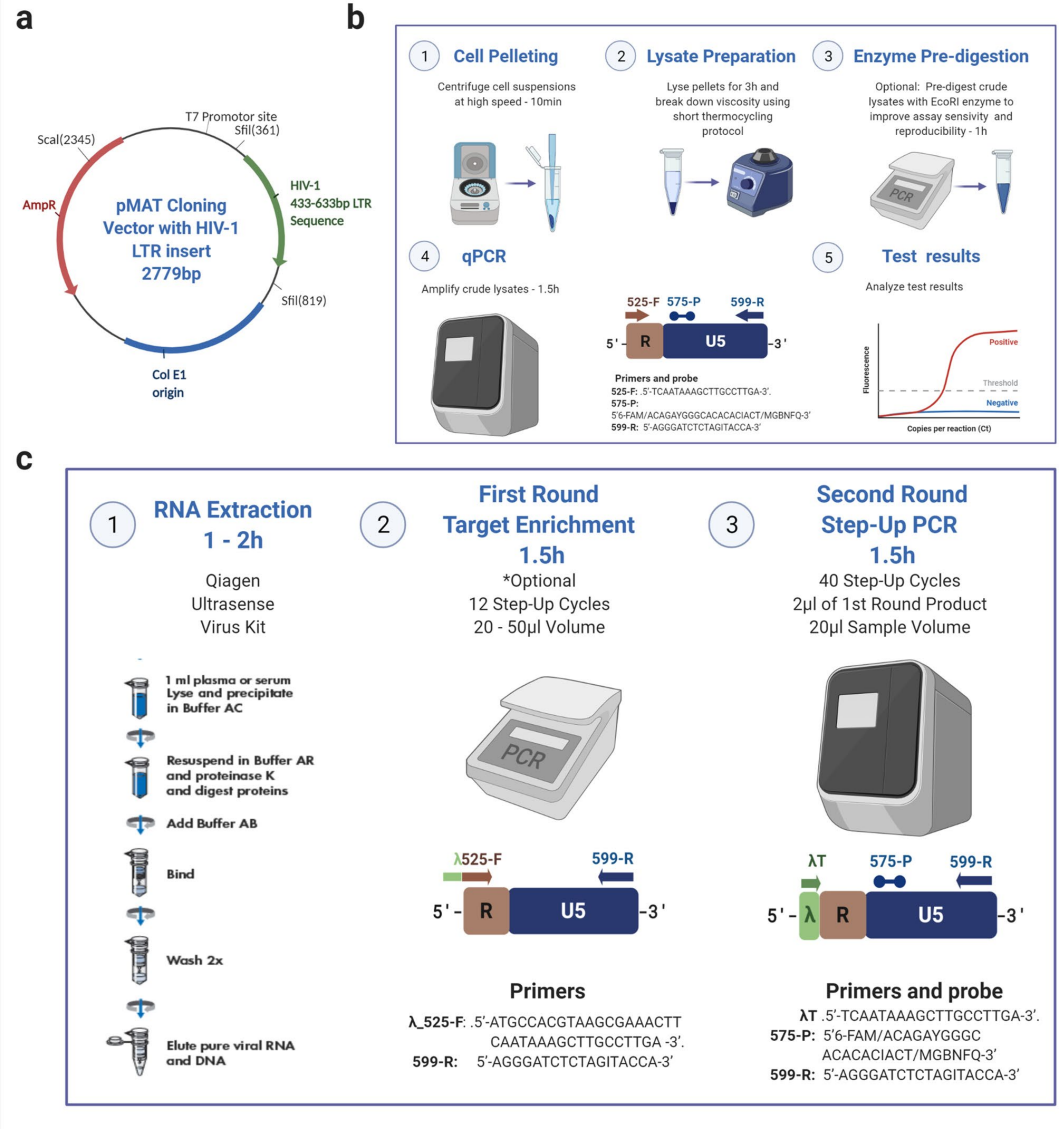
Revised qPCR and RTqPCR quantification assay synthetic standard and formats. Created with BioRender.com. (**a**) Synthetic DNA plasmid template containing the 433–633 HIV-1 LTR insert was linearized by *Sca*I and used for standard curve generation in the HIV-1 LDAs. RNA transcripts from the linearized DNA plasmid were made using the T7 promoter site and were used as HIV-1 RNA controls. (**b**) Protocol for Non-nested qPCR on crude DNA lysates. (**c**) Protocol for semi-nested RT-qPCR on RNA extracts.

**Figure 2:**
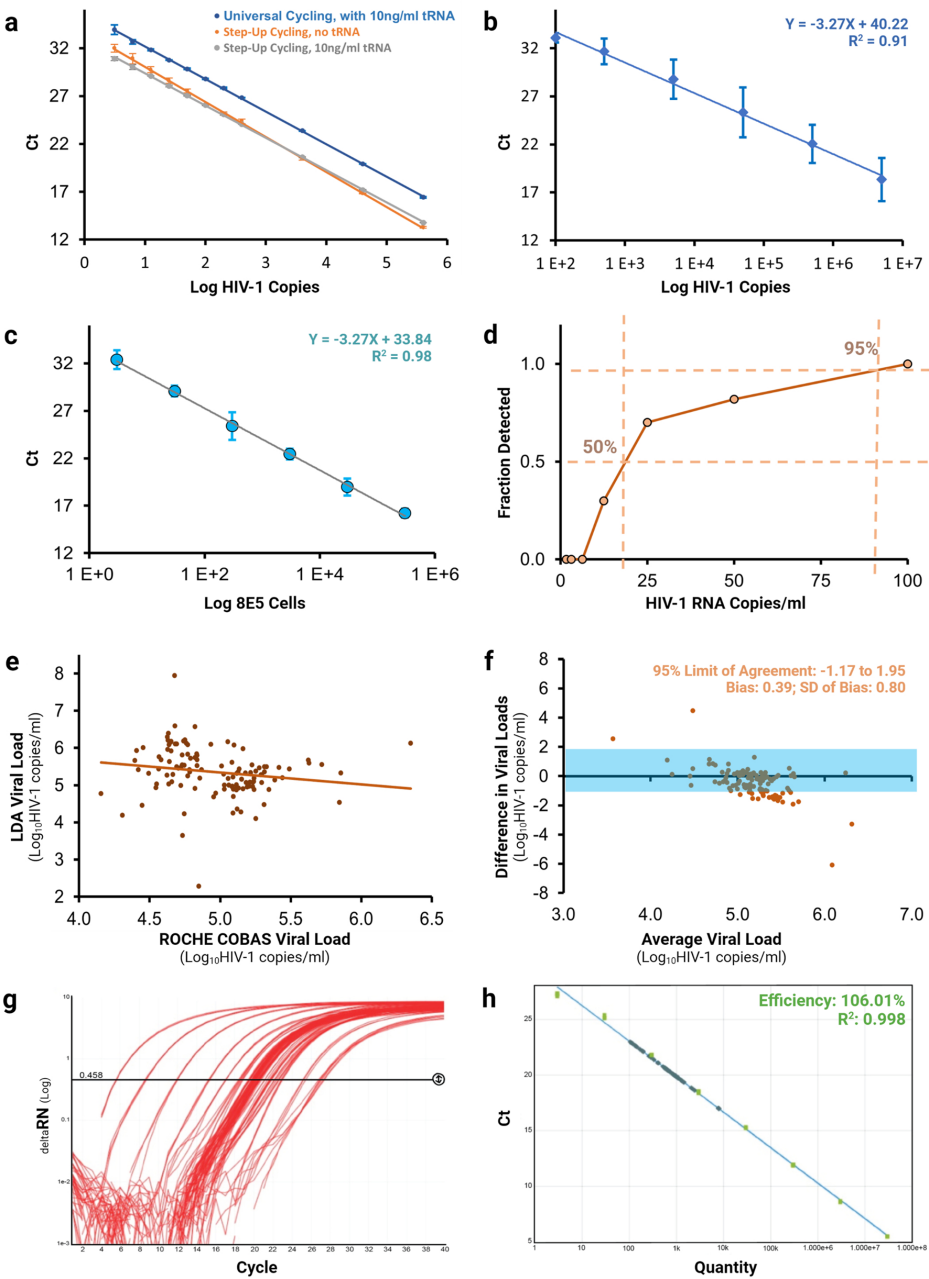
Optimization and Validation of Revised LTR-based qPCR and RTqPCR Assay Formats. (**a**) Optimization of the initial 496F/546P/633R primer–probe set for total HIV-1 DNA detection using universal cycling with tRNA; step-up cycling with no tRNA or step-up cycling with 10 ng/ml tRNA. Geometric means and standard errors of means are plotted for 3–12 replicates per sample. (**b**) Evaluation of the linearity of the RT-qPCR format of the 525F/574P/599R revised assay for detection of HIV-1 RNA on an AcroMetrix HIV-1 RNA Linearity Panel. (**c**) Demonstration of the linearity of the qPCR format of the revised HIV-1 laboratory developed assay on dilutions of crude lysates of 8E5 cells. (**d**) Plasma viral load limit of detection (LOD) determination of the RTqPCR format of the revised laboratory developed assay by probit regression of serial dilutions of EDTA plasma spiked with an HIV-1 AcroMetrix quantification standard near the cut-off level of the assay. The percentage of specimens detected at each copy level is indicated, and the 50% and 95% detection level extrapolated from the curve. Three replicates were used for 100 copies/ml while 10 replicates were used for the other dilutions. (**e**) Correlation in RNA plasma viral load measurements obtained by the revised laboratory developed assay and the Roche Cobas AmpliPrep/Cobas TaqMan HIV-1 test v2.0 assay. The analysis was performed on 127 high-titer HIV-1 spiked plasmas from the External Quality Assurance Program Oversite Laboratory (EQAPOL) and the US Military’s HIV Research Program (USMHRP). (**f**) Bland–Altman plot showing the difference between the measurements obtained by the revised laboratory developed assay and the Roche Cobas AmpliPrep/Cobas TaqMan HIV-1 test v2.0 assay. The analysis was performed on 127 high-titer HIV-1 spiked plasmas from the External Quality Assurance Program Oversite Laboratory (EQAPOL) and the US Military’s HIV Research Program (USMHRP). (**g**) A typical set of assay amplification curves obtained using the revised laboratory developed assay. (**h**) A typical standard curve obtained using the revised laboratory developed assay. NB: Graphs (**a**) to (**f**) were produced using Microsoft Excel. Graphs (**g**) and (**h**) were produced using the Thermofisher Cloud Platform Standard Curve Application. The final figure was created using BioRender.com

Assay optimization of the revised oligonucleotides was performed on a variety of instruments and finalized on a QuantiStudio 3™ thermocycler (ThermoFisher Scientific) using a major groove binding (MGB) probe containing a non-fluorescent quencher. The MGB group at the 3′ end of the probe increased its melting temperature (*T*_m_) while the NFQ component of the probe quenched the signal from the 6FAM fluorescent dye before hydrolysis, in a manner that resulted in an even lower background signal than a non-NFQ probe. The finalized protocol as described in the methods resulted in a greatly improved assay with a linear dynamic range of over 7 logs (Figs. 1, 2).

### PCR reagents and protocols

Generally, the step-up cycling protocol using 10 ng/ml tRNA provided greater sensitivity, linearity and precision when compared to universal cycling conditions (Fig. 2a; Table 2).

#### Real-time quantitative PCR (qPCR) protocol for DNA targets

When reaction volumes were decreased to 20 µl and maximum ramp rates or fast cycling was used, the PCR Biosystems Universal Probe (FAST) kit performed better than the QuantiFast™ Probe (Qiagen) and Kapa Probe Fast (Merck), providing a significant time and cost saving from the previously used Qiagen QuantiTect Probe kit.

#### Reverse transcriptase quantitative PCR (RT-qPCR) protocol for RNA targets

##### Standard non-nested RTqPCR

The standard non-nested format of the RTqPCR or viral load assay was adapted to the 1-Step Go mastermix (PCR Biosystems). It performed better than the EXPRESS One-Step SuperScript kit (ThermoFisher Scientific) and as well as the Superscript III/platinum kit when reaction volumes were decreased to 20 µl and maximum ramp rates or fast cycling was used. This provided significant time and cost savings.

For samples with target concentrations below 2 copies per µl, using a 50 µl final reaction volume and “touch up” cycling as recommended by the manufacturer, increased assay sensitivity tenfold with the 1-Step Go mastermix (PCR Biosystems).

##### Semi-nested RTqPCR for sample enrichment

The utilization of a semi-nested assay format allowed for target enrichment of limited samples and further improved assay sensitivity but was associated with more false positive control samples and failed assay runs due to cross-contamination.

### Assessing laboratory developed assay performance

#### Confirmation of correct sequence amplification

Gel electrophoresis of the qPCR products from the revised LDA produced bands of the expected size of 74 bp (599–525 = 74 bp). Sequencing of the bands confirmed the amplification of the expected region.

#### Assay linearity, lower limit of detection (LLOD) and lower limit of quantification with 95% confidence (LLOQ95)

A linear relationship was obtained for the qPCR format of the revised LDA against 8E5 cell DNA over a range of 10e1–10e7 input copies of HIV-1. This format of the assay was capable of reproducibly detecting the equivalent of a single infected cell (Fig. 2b, c; Table 3).

**Table 3:** Precision of assay as measured using dilutions of crude lysates of 8E5 cells.

Cell numbers	No. of replicates	Mean	SD	CV (%)
3	30	32.38	0.99	3.1
30	15	29.08	0.55	1.9
300	15	25.39	1.46	5.8
3000	15	22.44	0.56	2.5
30,000	15	18.97	0.91	4.8
300,000	15	16.21	0.47	2.9

Evaluation of the performance of the RT-qPCR format of the revised LDA showed good linearity, accuracy, and precision over a range of 100 to 5 × 10e6 copies of HIV-1 RNA/ml (R^2^ of 0.91) when evaluated on the AcroMetrix HIV-1 linearity panel (ThermoFisher Scientific) (Fig. 2b, c). The lower limit of detection (LLOD) was determined by probit regression on serial dilutions of EDTA plasma spiked with an HIV-1 AcroMetrix quantification standard near the cut-off level of the assay. The LLOD was found to be 88 copies/ml at the 95% level and 17 copies/ml at 50% (Fig. 2d).

#### Intra-assay and inter-assay reproducibility and precision

The precision of the assay was evaluated on serial dilutions of lysates of 8E5 cells testing over 15 replicates at 300,000 to 30 cell equivalents, and 30 replicates at 3 cell equivalents. Excellent reproducibility was observed at all dilutions, with the inter-assay coefficient of variation (CV) ranging from 1.9 to 5.8% and a CV of 3.1% at the 3 input copy level. Intra-assay variability comparing results obtained on different days was 3.7 ± 2.5% (Table 3).

#### Assay specificity for HIV-1

The assay was highly specific as demonstrated by a lack of signal in EDTA plasma from 10 uninfected donors (Biological Specialty Corporation) and a lack of reactivity with plasma from individuals infected with Influenza, Parainfluenza, RSV, Adenovirus, Human Metapneumovirus, Coronavirus, HBV, HCV, EBV, HSV-1, CMV, VZV, Parvovirus, HIV-2A and HIV-2B (Table 4).

**Table 4:** Specificity of assay on plasma samples from individuals infected with other viruses or from uninfected individuals.

Virus	Source	Result
Influenza Virus	CAP ID2-A 2012	TND
Parainfluenza Virus	CAP ID2-A 2012	TND
RSV	CAP ID2-A 2012	TND
Adenovirus	CAP ID2-A 2012	TND
Human Metapneumovirus	CAP ID2-A 2012	TND
Coronavirus	CAP ID2-A 2012	TND
HBV	Affymetrix-Valiquant	TND
HCV	Affymetrix-Optiquant	TND
EBV	Zeptometrix	TND
HSV-1	Zeptometrix	TND
CMV	Zeptometrix	TND
VZV	Zeptometrix	TND
Parvovirus	Zeptometrix	TND
HIV-2A	NIH Reagent Repository	TND
HIV-2B	NIH Reagent Repository	TND
Negative Plasma	BSC	TND
Negative Plasma	BSC	TND
Negative Plasma	BSC	TND
Negative Plasma	BSC	TND
Negative Plasma	BSC	TND
Negative Plasma	BSC	TND
Negative Plasma	BSC	TND
Negative Plasma	BSC	TND
Negative Plasma	BSC	TND
Negative Plasma	BSC	TND
Negative Plasma	BSC	TND
Negative Plasma	BSC	TND

*TND target not detected.

The assay was determined to be 95.76% accurate with 100% sensitivity and 93.24% specificity on 44 HIV-1 positive and 74 HIV-1 negative crude cellular lysates. The samples for this component of the validation were derived from 74 HIV-1 negative donors from IAVI’s Protocol L (formerly the International AIDS Vaccine Initiative). They comprised of 26 male and 46 female participants and 2 donors with unknown gender. The group contained 12 chronically infected protocol L donors (8 male and 4 female) and 32 cART-suppressed male HIV-1 positive donors from the London St. Stephens Trust. The protocol L donors come from regions of Rwanda and Kenya where HIV-1 subtype A is predominant, followed by D, C and G while the London participants were all confirmed as subtype B (Supplementary Table S5). The positive predictive value of the assay was 89.80% while the negative predictive value was 100% with this sample set (Table 5).

**Table 5:** Specificity of the revised 525F/575P/599R laboratory developed assay on crude PBMC lysates from HIV-1 infected or uninfected individuals.

Patient HIV infection status	HIV-1 assay results			Sensitivity (%)	Specificity (%)	Positive predictive value (%)	Negative predictive value (%)	Accuracy (%)
	Positive	Negative	Total					
Infected	44	0	44					
Uninfected	5	69	74					
Total	49	69	118	100 (91.96–100)	93.24 (84.93–97.77)	89.80 (79.06–95.35)	100	95.76 (90.39–98.61)

*See Supplementary Table S5 for Donor Characteristics.

^a^Calculations computed using https://www.medcalc.org/calc/diagnostic_test.php.

^b^Values in parentheses are the 95% confidence intervals.

#### HIV-1 cross-subtype specificity

The revised LDA accurately detected and quantified 19 out of the 20 problem strains in the preliminary diversity panel (95%) but missed the CRF01_AE strain KC596065 (Table 2). The ability of the assay to provide accurate quantitative measurements of plasma viral load over a wide range of HIV-1 subtypes was then evaluated on a panel of 127 isolates from the External Quality Assurance Program Oversite Laboratory (EQAPOL) and the US Military’s HIV Research Program (USMHRP) spiked into plasma samples. The assay detected all but one of the additional virus strains in the panel (Fig. 2f). It also failed to detect a 2004 subtype C isolate from China (AY713414). This represented a 99.2% detection rate.

A Bland–Altman plot showing the difference between the measurements obtained by the revised LDA compared to viral load values obtained by the Roche Cobas AmpliPrep/Cobas TaqMan HIV-1 test v2.0™ assay shows a tight relationship with an R^2^ of 0.03, representing parallel performance (Fig. 2d), with a slight but statistically insignificant under-quantification bias towards the laboratory developed assay (0.388) (Fig. 2e, g).

**Figure 3:**
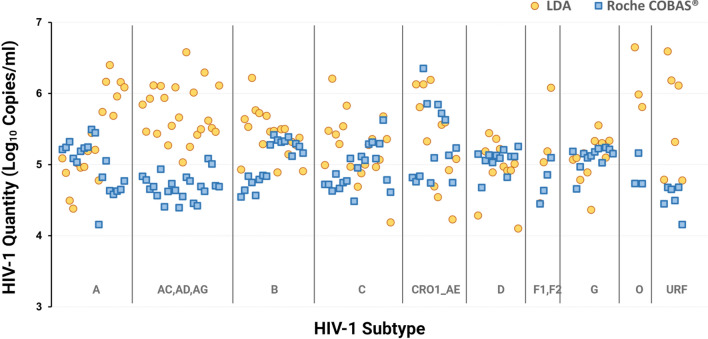
Comparison of HIV-1 viral load measurements by HIV-1 subtype determined by the revised laboratory developed assay (LDA) and the Roche Cobas AmpliPrep/Cobas TaqMan HIV-1 test v2.0 assay.

The relationship between the LDA and the Roche assay by individual isolates as sorted by subtype is shown in Fig. 3, with the raw data in Supplementary Table S3. Additional, more detailed, raw data files have been deposited in a center for open science database and can be viewed via this link - https://osf.io/m9yne/?view_only=47cbcfd3d39b4a89a0d758a474441e38.

## Discussion

A highly sensitive, specific, and robust quantitative PCR assay, targeting the HIV-1 LTR, was developed for use in research and for quantifying viral levels in HIV-1 infected individuals. Different assay formats and protocols were developed including a traditional RT-qPCR format for viral load determination and a cell-based DNA format that can be used to monitor residual levels of viral nucleic acids in treatment-suppressed individuals (see Supplementary Table S4). The assay design included selection of primers, probes and amplification conditions to allow sensitive detection of all major HIV-1 subtypes worldwide, including group M subtypes A (East Africa), C (Southern Africa, India, Nepal), D (Eastern and Central Africa), CRF01_A/E (Thailand), and CRF02_A/G (West Africa and Central Europe) and Group O.

A variety of thermocyclers and master mixes were evaluated in the development process of this assay. The final optimized version used the thermofisher Quantistudio 3.0 thermocycler which has various customizable features. Adapting the assay to alternative thermocyclers may require some additional tweaking or optimization but should maintain the same basic principles as described in this manuscript. The adaptation of the MGB group at the 3′ end of the probe allowed for lowering the melting temperature (*T*_m_) while the NFQ component of the probe quenches the signal from the 6FAM fluorescent dye before hydrolysis, in a manner that results in an even lower background signal than a non-NFQ probe.

Despite initially selecting the most promising oligonucleotide sequences that had been reported in the literature as being broadly cross-subtype specific, assessment of the Brussel 2005 assay with a Friedrich 2010 internal reverse primer, which performed exceptionally well on a synthetic template, found that it accurately quantified only 7 of 20 (35%) problem strains of HIV-1 viruses tested in a cross-subtype EQAPOL diversity sub-panel (Table 2). Interestingly, alignment of these oligonucleotide sequences against the published sequences of the panel members did not reveal any mismatches in the strains that were not detected by the assay. Alignment of these oligonucleotides against the compendium database, however, revealed several mismatch issues, particularly in subtype AE and O (Supplementary Fig. S2; Supplementary Table S2). A similar alignment of the revised LDA oligonucleotides showed significantly better homology across a diverse range of HIV-1 subtypes and thus detected and quantified them better (Supplementary Table S2; Supplementary Fig. S2). This was unexpected and indicates that in-silico predictions of cross-subtype specificity based simply on sequence alignments can sometimes be misleading^25^.

As stated, before designing the revised oligonucleotide set, a more rigorous alignment analysis of the Brussel/Friedrich oligonucleotide sequences was conducted against the current edition of the LANL compendium database (Supplementary Fig. S1b, c). The compendium database is comprised of 37 highly curated sequences from major HIV-1 group M subtypes (four sequences per subtypes); 32 CRFs and other reference sequences and 11 subtype N, O or CPZ. The database is designed to represent the diversity of the sequences present within the entire HIV-1 database but allows quicker and more accurate analysis of variation as repetition and poor sequence information is eliminated. The alignments against the compendium database more accurately reflected assay performance and identified multiple mismatch issues, particularly in subtype AE and O (Supplementary Table S2). The revised LDA was therefore designed against the compendium database and then verified against the entire database. It corrected the mismatch issues found in the Brussel/Friedrich oligonucleotides and incorporated a wobble and Inosine, a modified base, in two locations. As already stated, the resulting assay performed much better than the Brussel/Friedrich assay on a large diverse panel of HIV-1 strains.

To determine whether the discrepancy between general sequence alignment versus the compendium alignment was a common phenomenon, we re-analyzed oligonucleotide sets reported in the literature as having good HIV-1 cross-subtype specificity against the compendium database; using the same method we adopted to design the revised LDA. When the assays reported by Vandergeeten 2014 and van der Sluis 2013 as highly sensitive and broadly cross reactive, for example, were analyzed against the compendium database, their base-pair identity statistics did not look as favorable as indicated in the original publications. (Supplementary Table S2). Additionally, Rutsaert et al. recently conducted an in-depth analysis of 20 total HIV-1 DNA assays reported in the literature as having good cross-subtype specificity including the afore mentioned Vandergeeten 2014, and van der Sluis 2013 assays, against an HIV-1 subtype diversity panel comprised of 30 samples. We aligned the oligonucleotides from the two best-performing assays from their analysis—Schvachsa 2007 and Viard 2004—against the compendium database and found that these alignment statistics were also not as good as the revised LDA (Supplementary Table S2)^26–28^.

The LTR region of HIV-1 is particularly problematic for accurate assay design because sequencing enzymes become error-prone and drop off at the end of genomes. Indeed, despite all the sequences being classified as “complete”, there are many sequences with only partial or no information within the LTR region, even in the carefully curated compendium database. As can be seen from the sequence alignments in Supplementary Table S2, the compendium database had 21% less sequence information in the LTR regions where sequence information becomes scarcer—i.e., towards the ends of the genome—compared to the sequence information available for upstream regions of the virus. Of all 198 sequences in the compendium database, 101 (51%) had information in the 633R primer region for example, compared to 65 (32.3%) in the 525F primer region and 53 (26.7%) in the 496F primer region of the assay.

We recommend therefore, that future HIV-1 PCR assay design be based on the compendium database rather than the entire HIV-1 database if good cross-subtype specificity is required. We suggest that there is in fact a need for a more comprehensive compendium database specifically for assay design and evaluation. Ultimately, assay validation and optimization must still rely on using independently assembled, large, diversity panels, that include recent strains of the virus, such as the EQAPOL panels, to gain a more accurate picture of the performance of in-house assays in real field situations^29^.

The variability of the HIV-1 genome makes it very challenging to find three well-conserved regions of 25–35 nucleotides (two for the primers and one for the internal probe) within 200 base pairs of each other, for use with TaqMan PCR. The rationale behind the step-up or touch-up amplification methods was to allow for less stringent annealing at lower initial temperatures, followed gradually by higher more stringent annealing temperatures. The addition of yeast tRNA to PCR master-mixes and sample diluents significantly improved assay efficiency, quantification sensitivity and precision at low viral levels, such as are found in treated subjects.

The semi-nested format of the assay, with a tag on the forward primer in the first round and a primer against this tag in the second round, allowed for the use of only three regions and increased the chance of good cross-subtype coverage. This format allows for target enrichment and is recommended for limited samples. Rigorous cleanliness and great care must be taken to avoid cross-contamination with the semi-nested assay format. The use of a “tag” on one of the primers coupled with less stringent initial annealing temperatures, allowed for amplification across regions of incomplete homology which increased the chance of good cross-subtype coverage. The tag subsequently allowed amplification of only specific amplicons from the first round by reducing nonspecific amplification of any primer-dimers or nonspecific artefacts generated in the primary PCR thus maintaining PCR specificity.

The use of crude DNA lysis makes the qPCR format of the assay easy and affordable. It maximizes assay sensitivity by preventing the unnecessary loss of precious sample through more complicated DNA extraction procedures. Using primers located in close proximity to the ones in our assay, Avettand-Fènoël et al. (2009), demonstrated successful quantification of HIV-1 DNA in whole blood samples for early diagnosis even in masked primary infection in newborns whose mothers have received cART. This and other studies indicate that intracellular DNA quantification could serve as a more accessible disease treatment-monitoring tool, particularly in resource-limited settings. This format of the assay was also developed as an alternative to the QVOA assay for analyzing residual latent virus. This sample type, however, is prone to false positives. See Supplementary Table S4 for a summary of the different formats, their recommended use, their advantages and disadvantages.

It was very important to assess the specificity of the crude DNA lysate qPCR format of the assay on a panel of HIV-1 negative and positive PBMC lysates. While all the HIV-1 positive PBMC samples, including 32 viral-load undetectable samples, were quantified by the assay, 5 out of 74 negative samples were scored as positive. It may be possible that some of the individuals who had been scored as negative had been at early stages of HIV infection. The HIV screening process involved two rapid antibody tests with any discordant results confirmed by ELISA. Only HIV positives receive a viral load test, which would detect infection by several days up to two weeks earlier. However, since the PCR assay was run in a laboratory setting that is not specialized for molecular work, we cannot rule out the possibility that the false positives observed are due to low level cross-contamination by HIV-1 tissue culture or high-titer HIV-1 plasmid clone preparation conducted in the sample preparation areas. Despite great care to decontaminate the workspaces, background contamination is an inherent problem within the current setting. More rigorous analysis in a highly controlled environment may be required to decrease the rates of false positives.

Detection of very low levels of residual viral sequences as may be present in cART-treated individuals with undetectable viral loads has important applications for predicting re-emergence of infectivity or evaluation of approaches to viral cure. An important limitation of PCR-based assays such as those described here, is that they cannot differentiate between intact viral genomes and defective, deletion genomes which occur at far greater frequency and persist for a long time. More recent PCR assays such as the Intact Proviral DNA Assay (IPDA) were designed to distinguish between and separately quantify intact and defective proviruses^30^. but are far more expensive, very difficult to standardize and implement for routine testing, and far less sensitive than PCR-based assays such the revised LDA reported here (Supplementary Table S4).

The semi-nested version of the assay allows for far greater sensitivity for use in research applications but is prone to cross-contamination which may limit its routine application in many laboratories, especially in resource limited settings where separate PCR containment areas may not be so readily available (Supplementary Table S4).

The revised LDA failed to detect two strains that the Roche assay detected. Commercial assays commonly target more than one conserved region of the HIV-1 genome to ensure maximal cross-subtype specificity. The LDA could thus be combined with an assay against conserved regions of the integrase or polymerase gene to improve its cross-subtype specificity. Overall, the revised LDA has very good performance characteristics and presents a cost-efficient and viable viral detection and monitoring tool for clinical research use, particularly in resource-limited settings where the need is greatest^1,3,31,32^. As previously indicated, additional detailed raw data files corresponding to Figures 2 and 3; Tables 1, 3, 4 and 5 and Supplementary Table 2, have been deposited in a "center for open science" database and can be viewed via this link - https://osf.io/m9yne/?view_only=47cbcfd3d39b4a89a0d758a474441e38. Please note, as previously stated, that this technology is the subject of a priority patent application. Any parties wishing to commercialize these assays or variations thereof, must contact Imperial College London via enterprise@imperial.ac.uk citing reference number 10579.

## Conclusions

Evaluation of the revised 525F/575P/599R LDA in accordance with performance guidelines for monitoring viral load of HIV-1 infected individuals^33,34^ demonstrated a high degree of precision, specificity, sensitivity, and linearity across a wide dynamic range. The LDA is highly sensitive with good cross-subtype specificity and has the potential to play an important role in HIV-1 research and in improving clinical outcomes if used correctly^21^. Using semi-nested qPCR, for target enrichment, allows for highly sensitive detection, when compared to non-nested qPCR and increases the likelihood of detecting ultra-low levels of HIV-1 within samples. This assay provides a convenient, sensitive, specific, and reproducible measure of HIV-1 viral RNA in plasma and HIV-1 viral RNA and DNA in the PBMCs of patients under anti-HIV therapy. The assay is suitable for monitoring the efficacy of therapeutic strategies and for measurement of viral persistence in support of studies aimed at testing the efficacy of vaccines, antiretroviral combinations, and HIV-1 eradication strategies.

## Materials and methods

The Polymerase Chain Reaction or PCR forms the basis for HIV-1 viral measurement in blood or other fluids and within cellular compartments of the human body. The method, as we have used it, targets specific regions of HIV-1 nucleic acids and amplifies them exponentially with detection via a fluorescent read-out.

### Assay design

As previously stated, oligonucleotide sequence names were denoted by the first nucleotide of their HXB2 reference sequence base position and an “F” for forward primer, “P” for probe or “R” for reverse primer. The main oligonucleotide sets studied during this project were also denoted by the first author and their year of publication e.g., Brussel 2005. Probes were initially prepared in a TaqMan PCR format with doubly quenched Iowa-Zen chemistry (www.idtdna.com) as this had previously been found to provide good sensitivity in our laboratories.

### Previously reported laboratory developed assays

Initial selection of assay oligonucleotides was based on a literature review and assessment of candidate oligonucleotides for broad cross-subtype specificity against all available sequences in the United States Los Alamos National Medical Library’s (LANL) HIV-1 database (www.hiv.lanl.gov) using the QuickAlign tool. The full list of oligonucleotide sets selected for initial evaluation are shown in Supplementary Table S1.

### Revised laboratory developed assays

To improve cross-subtype specificity, a revised oligonucleotide set—525F/574P/599R, was designed by using AlleleID software v7.0 (PREMIER Biosoft, San Francisco, CA, USA) (http://www.premierbiosoft.com/bacterial-identification/index.html) against an alignment of the relatively homologous region running from 433 to 633 base pairs of the HIV-1 long terminal repeat (LTR) from the LANL HIV-1 compendium database (https://www.hiv.lanl.gov/content/sequence/HIV/COMPENDIUM/compendium.html). The oligonucleotide selection was finalized by using the Integrated DNA Technology online oligoanalyzer tool (https://eu.idtdna.com/pages/tools/oligoanalyzer) to select the sequences that provided the best in silico assay parameters from the possible permutations. Analysis of the finalized sequences against the compendium database showed very few mismatches (Supplementary Table S2; Supplementary Fig. S2). These mismatches were accounted for by replacements with a wobble base and inosine modified bases at relevant positions (Table 1, Supplementary Fig. S2).

To allow maximum utility, we developed the HIV-1 assay in both a standard and a semi-nested format, using both DNA and RNA targets. The semi-nested format allows target enrichment of limited samples and thus increases assay sensitivity. The RTqPCR format for quantification of HIV-1 RNA in fluids, including plasma viral load, was optimized, and validated using synthetic plasmid templates and WHO-recognized international standards. The qPCR format, for quantification of total nucleic acids (i.e., RNA and DNA) in cellular compartments, was optimized and validated using synthetic plasmid templates and crude lysates from 8E5 cells, a recognized cell-line standard.

### Synthetic target plasmid templates for assay development

#### HIV-1 LTR DNA template

The following sequence from 433 to 633 bp of the HIV-1 LTR was used: CAGCTGCTTTTTGCCTGTACTGGGTCTCTCTGGTTAGACCAGATCTGAGCCTGGGAGCTCTCTGGCTAACTAGGGAACCCACTGCTTAAGCCTCAATAAAGCTTGCCTTGAGTGCTTCAAGTAGTGTGTGCCCGTCTGTTGTGTGACTCTGGTAACTAGAGATCCCTCAGACCCTTTTAGTCAGTGTGGAAAATCTCTAGC.

The fragment was cloned into a pMA-T vector at the Sfil cloning site by the Life Technologies GeneArt Service (ThermoFisher Scientific). Plasmid DNA was purified from transformed *E. coli* K12 (dam + dcm + tonA) (Fig. 1a), linearized using a single-cutter ScaI-HF® Restriction Enzyme (New England BioLabs, Ipswich, MA, USA) and purified using a PureLink™ Quick Gel Extraction Kit (Invitrogen/ThermoFisher Scientific, Waltham, MA, USA).

#### Extraction control DNA template

A DNA plasmid incorporating a previously described SCP1 random probe sequence—CTGGGTAGAGTAGTCACAGAATGCG^35^ flanked by the 496F and 622R HIV-1 primers (Supplementary Table S1) was synthesized, via the ThermoFisher GeneArt service, using a manufacturer-selected cloning vector.

#### HIV-1 LTR and extraction control RNA templates

RNA transcripts of the linearized DNA templates were synthesized using the MEGAscript T7 Transcription Kit (Ambion/ThermoFisher Scientific). The size and purity of the products was confirmed by gel electrophoresis. The gel-purified transcripts were quantified using a NanoDrop™ 1000 spectrophotometer (ThermoFisher Scientific).

#### HPRT-1 reference gene cDNA template

A template encoding the HPRT region targeted by an in-house RT-qPCR reference gene assay–TGACACTGGCAAAACAATGCAGACTTTGCTTTCCTTGGTCAGGCAGTATAATC CAAAGATGGTCAAGGTCGCAAGCTTGCTGGTGAAAAGGACC^36^ was synthesized via the ThermoFisher GeneArt service, using a manufacturer-selected cloning vector. RNA sample data was considered valid if the reference gene could be detected regardless of whether a positive HIV-1 signal was detected.

#### Synthetic template copy number calculation and standard generation

The copy number/µl for each synthetic template stock solution was determined from its molecular weight and Avogadro’s constant using an online copy number calculator (http://www.endmemo.com/bio/dnacopynum.php). The stocks were diluted to working solutions of either 1e8 or 3e8 copies/µl in Tris/EDTA pH 8.0 (TE) buffer for DNA or DEPC-treated water for RNA respectively. The working solutions were divided into 5 µl single-use aliquots in PCR tube strips and stored at − 80 °C. Single aliquots were broken off the strips and thawed before each use. Ten-fold standard curve dilutions ranging from 3e7 to 3 viral input copies were generally run in triplicate. More replicates were run at the lowest dilutions when determining the assay lower limit of detection (LLOD), lower limit of quantification (LLOQ), precision, and reproducibility.

### Ethics

For the Kenyan donors from IAVI’s protocol L, ethical approval for sample collection and processing was granted by the Kenyatta National Hospital /University of Nairobi ethics and research committee (KNH/UON-ERC); study reference P69/03/2011. For the Rwandese donors from IAVI’s protocol L, ethical approval for sample collection and processing was granted by the Rwanda National Ethics Committee (RNEC) Research Ethics Committee; study reference No.1023/RNEC/2020.

For the donor samples from the London St. Stephen’s Trust, ethical approval for sample collection and processing was granted by the NHS London-Chelsea Research Ethics Committee; study reference 96.ND14, RREC1108. Informed written consent was provided by all participants prior to obtaining each sample, and clinical data was collected as part of standard care.

The samples from the EQAPOL and AIDS Reference Reagent Program repositories have all been anonymized with no links back to the individual samples. They are exempt from regulations concerning human samples.

### Sample preparation

#### Crude cellular DNA lysates

For PBMC lysate preparation, cryovials containing frozen cells were thawed in a water bath at 37 °C for approximately 2 min until only a small ice crystal remained. The cells were then transferred into 50 ml pre-warmed RPMI containing 10% fetal calf serum, 10 mM HEPES, 2 mM l-glutamine, 1 mM sodium pyruvate and 1× penicillin–streptomycin. Cells were pelleted at 250 g for 10 min, supernatants decanted, and cells resuspended in 1 ml PBS. Cell concentrations were determined using a Vi-cell counter (Beckman Coulter Instruments, Inc.). Cells were then made up to 1e6 cells per ml in PBS, divided into 1 ml aliquots in sample tubes and pelleted at 2500 g for 5 min. Supernatants were aspirated twice by pipetting to ensure a very dry pellet before cell lysis. For 8E5 cell lysate preparation, frozen pre-made pellets containing 1e7 cells were used (see section titled “Cellular Standards for DNA qPCR”).

A lysis buffer was prepared using 10 mM Tris HCL pH8 (Ambion), 50 mM KCl (Ambion) and 0.4 mg/ml Proteinase K (Qiagen) in molecular biology grade water. Forty-two µl of lysis buffer was added to the cell pellets and vortexed for 10–15 s. Cells were lysed for 3 h at 55 °C. To reduce DNA viscosity and break-up clumps, the following cycling protocol was then used: 65 °C for 1 min, 96 °C for 2 min, 65 °C for 4 min, 96 °C for 1 min, 65 °C for 1 min, 96 °C for 30 s. Finally, lysates were incubated at 95 °C for 15 min to ensure complete inactivation of the proteinase K. DNA was quantified using a NanoDrop 1000 spectrophotometer (ThermoFisher Scientific) and genome copy number/µl determined by molecular weight and Avogadro’s constant using an online copy number calculator (http://www.endmemo.com/bio/dnacopynum.php).

To improve sensitivity and precision at the lowest target concentrations, DNA lysates were pre-digested with an EcoRI-HF enzyme (New England BioLabs, Ipswich, MA, USA), according to the manufacturer’s instructions, before amplification (Fig. 1b). The EcoRI-HF enzyme was chosen because it did not cut DNA within the LDA target region.

#### Plasma RNA extraction

The QIAamp UltraSens Virus Kit (Qiagen) was used for viral RNA extraction from plasma samples with the following modifications: -1 ml of each HIV-1 spiked EDTA plasma sample was used; 3e6 input copies of the extraction control template was added to the spin columns along with the lysed and proteinase-digested samples; the lysate and AW1 buffer were centrifuged at 8000 g; all subsequent centrifugation steps were at 20,000*g*; only one final RNA elution step was performed using 30 µl AVE buffer following a 5-min incubation at room temperature.

#### Cellular standards for DNA qPCR

8E5 cells are an established lymphocyte cell line that contain a single copy of integrated HIV-1 DNA genome per cell^37^. The 8E5 cells were obtained from the AIDS reagent program (https://www.hivreagentprogram.org Cat No. ARP 95) and passaged 3 times in RPMI containing 10% fetal calf serum, 10 mM HEPES, 2 mM l-glutamine, 1 mM sodium pyruvate and 1× penicillin–streptomycin at 37 °C, 5% CO_2_. Cells were then pelleted at 250 g for 10 min, washed in PBS and divided into 1 ml aliquots of 1e7 cells per cryovial. Cells were pelleted at 250 g for 10 min then resuspended in 1 ml of ice-cold Fetal Calf Serum containing 10% Dimethyl Sulfoxide (DMSO). The cryovials were frozen down to − 80 °C overnight in a rate-controlled Stratagene stratacooler and stored at − 150 °C in vapor phase liquid nitrogen.

To prepare cell pellets for the generation of PCR standard curves, an aliquot of each of the working stocks of the cells was thawed out as in the previous section when required and passaged 3–6 more times. The cell suspensions were then centrifuged at 250 g for 10 min, quantified using a ViCell Counter (Beckman Coulter Instruments, Inc.) and adjusted to 1e7 cells/ml in PBS. One ml aliquots of the cell suspensions were divided into cryovials and the cells were pelleted at maximum speed for 10 min in a benchtop microfuge. Supernatants were carefully removed twice by pipetting, and the dried cell pellets stored at − 80 °C until required.

#### Instrument and probe–chemistry optimization

A series of real-time PCR instruments including the Applied Biosystems 7500 Dx thermocycler, Applied Biosystems 7300 thermocycler, Applied Biosystems StepOnePlus thermocycler, Roche Light-cycler and finally the ThermoFisher QuantiStudio 3 thermocycler were evaluated with the assay. In all cases default manufacturer settings were used. The QuantiStudio 3 instrument and its manufacturer recommended Minor Grove Binding (MGB) TaqMan probe labeled with 6-CaboxyFlourescein at the 5′ end and a Non-fluorescent quencher (NFQ) at the 3′ (6FAM/MGBNFQ) provided the best performance.

#### PCR reagents and protocols

For initial primer evaluation, the QuantiTect Probe kit (Qiagen) for qPCR of DNA targets and the SuperScript III Platinum One-Step qRT-PCR Kit (ThermoFisher Scientific) for RNA targets, were used because they were the reagents commonly used in our laboratories.

To shorten assay time using fast-cycling, we evaluated the use of the following test kits and master mixes for qPCR:—the QuantiFast Probe (Qiagen); Kapa Probe Fast (Merck) ;or qPCRBIO Probe Mix Lo-Rox (PCR Biosystems); the EXPRESS One-Step SuperScript (ThermoFisher Scientific); and the qPCRBIO 1-Step Go Lo-Rox kit (PCR Biosystems), based on recommendations from PCR instrument manufacturers and colleagues.

To further increase assay sensitivity and reduce variability at low target concentrations, various concentrations of tRNA were added to the master-mix and optimized. A published “step-up” cycling protocol was employed to improve sensitivity and cross-subtype specificity^38^. “Step-up” cycling rescues PCR reactions where there are minor mismatches between the assay oligonucleotides and the target template species and serves to increase the sensitivity and precision for detection of HIV subtypes with minor sequence polymorphisms at low target concentrations.

#### Real-Time Quantitative PCR (qPCR) Protocol for DNA Targets

The final conditions selected for PCR amplification were the qPCRBIO Probe Mix Lo-Rox reagents (PCR Biosystems) with 600 nM Forward Primer, 600 nM Reverse Primer, 200 nM Probe (Table 2); up to 3e6 HIV input-copies of enzyme-digested target DNA; 10 ng/ml yeast tRNA (Sigma-Aldrich, MO, USA) added to the PCR master-mix, and a “step-up” cycling protocol with an initial activation step of 95 °C for 15 min, 3 pre-amplification cycles of 94 °C for 20 s, 52 °C (8 °C below the ideal annealing temperature) for 10 s and 60 °C for 1 min, followed by 40 amplification cycles of 94 °C for 20 s, 56 °C (4 °C below the ideal annealing temperature) for 10 s, 60 °C for 1 min. Fluorescent data was collected at the 60 °C amplification step of each of the 40 cycles (Fig. 1b). Cell copy number in DNA samples was calculated relative to an RNaseP TaqMan copy number reference assay (Applied Biosystems).

### Reverse transcriptase quantitative PCR (RT-qPCR) protocol for RNA targets

#### Standard non-nested RTqPCR

A standard, non-nested, RT-qPCR protocol was used to determine the LLOQ plasma viral load of the assay. The qPCRBIO 1-Step Go Lo-Rox kit (PCR Biosystems) was used with the same oligonucleotide and tRNA concentrations as in the qPCR protocol. As recommended by the manufacturer, an initial reverse transcription step of 50 °C for 10 min was followed by a “touch up” cycling protocol with an enzyme activation step of 95 °C for 2 min; 40 amplification cycles of 95 °C for 10 s, 52 °C → 60 °C* for 10 s and finally 72 °C for 1 min. The arrow and asterisk indicates that the annealing temperature was increased from 52 to 60 °C in equal increments across 40 cycles. Five µl of sample was used in a final reaction volume of 50 µl for maximum sensitivity.

#### Semi-nested RTqPCR for sample enrichment

To allow target enrichment of limited archival samples, a semi-nested RT-qPCR protocol was also developed (Fig. 1c). A bacteriophage lambda sequence—a foreign, non-human oligonucleotide—was used as a tag on the first-round forward primer, λ-525F, while a primer against the tag—λT (Table 2), was used in the second round of the PCR. For this semi-nested protocol, HIV-1 cDNA synthesis and first round amplification were conducted using an Applied Biosystems Veriti Thermal Cycler (Applied Biosystems/ThermoFisher Scientific) and the qPCRBIO 1-Step Go Lo-Rox kit (PCR Biosystems) with 600 nM λ_525F and 599R primers. A “step-up” cycling protocol was adopted with a reverse transcription step of 50 °C for 15 min followed by a polymerase activation step of 95 °C for 2 min; five amplification cycles of 95 °C for 20 s; 52 °C for 10 s and 60 °C for 1 min; followed by seven cycles of 95 °C for 20 s, 56 °C for 10 s, and 60 °C for 1 min. For second round amplification, 2 µl of the first-round product was used in the 20 µl second round amplification (10%v/v), with 600 nM λT forward primer, 600 nM 599R reverse primer and 200 nM 574P probe (Table 2). The same reagents, instrumentation and cycling protocol described for the qPCR of DNA targets were used.

The presence of RNA in sample extracts was confirmed using an in-house HPRT1 reference gene assay and the HPRT synthetic template described previously to generate the standard curves. The qPCRBIO 1-Step Go Lo-Rox Kit reagents (PCR Biosystems) and manufacturer recommended cycling parameters were used with 900 nM TGACACTGGCAAAACAATGCA HPRT Forward Primer, 900 nM GGTCCTTTTCACCAGCAAGCTHPRT Reverse Primer and 250 nM CTTCCTTGGTCAGGCAGTATAATC VIC/TAMRA Probe (TermoFisher Scientifc).

#### qPCR and RTqPCR run acceptance criteria

Each assay run included quantification standards, and a known positive sample or an AcroMetrix HIV-1 positive control or calibrator (ThermoFisher Scientific); and a known negative sample or an AcroMetrix HIV-1 negative control (ThermoFisher Scientific). Assay runs were considered acceptable if no target was detected in the non-template and negative controls, the standard curve had a slope of − 3.2 to − 3.5, and R^2^ values were > 0.95. Linearity was based on the slope and R^2^ of the standard curve and expressed as y = mx. The linear range of the assay was determined from the standard curves. Precision/reproducibility was expressed as a percentage of the coefficient of variability (CV), with a CV of less than 10% being acceptable. HIV-1 RNA target runs were only considered valid if a parallel HPRT1 reference gene assay was also valid and HPRT1 was detected in the samples, while HIV-1 DNA target runs were only considered valid if a parallel RNaseP reference gene assay was also valid and RNaseP was detected in the samples.

### Assessing laboratory developed assay performance

#### Confirmation of correct sequence amplification

To determine whether a single PCR product of the desired length and sequence was being produced by the assays, PCR products were run on a 1% Agarose gel (Fluka, NJ, USA). Bands were visualized using the SYBR Safe DNA Gel Stain (Invitrogen) and a Bio-Rad Molecular Imager Gel Doc XR + with Imager Lab Software (Bio-Rad Laboratories) and cut out on a safe Imager 2.0 (ThermoFisher Scientific). DNA was recovered using a QIAquick gel purification kit (Qiagen). Purified PCR products were sequenced by the Sanger sequencing service at Genewiz, Surrey, United Kingdom and confirmed proper amplification of the target sequence.

#### Assay linearity, lower limit of detection (LLOD) and lower limit of quantification with 95% confidence (LLOQ95)

Linearity of the HIV-1 LDA was evaluated in a non-nested format on triplicate samples of the AcroMetrix HIV-1 Quantification Panel (ThermoFisher Scientific) ranging from 5e2 to 5e6 copies/ml.

LLOD/LLOQ95 values were calculated based on detection of two-fold dilutions of negative EDTA plasma samples spiked with the 5e4 copies/ml Acrometrix standard (ThermoFisher Scientific) at dilutions ranging from 25 to 0.46 copies per ml using 10 replicates per data point.

#### Intra-assay and inter-assay reproducibility and precision

Cell pellet aliquots were thawed, lysed and quantified as previously described in the “Cellular Standard for DNA qPCR” section. Ten-fold serial dilutions ranging from 3e6 to 3 input cell equivalents per reaction were prepared in TE Buffer containing 100 µg/ml salmon sperm DNA (ThermoFisher Scientific).

Intra-assay reproducibility was evaluated on 3 separate batches of serially diluted 8E5 cell lysates tested by a single technician on 3 separate occasions, using the standard qPCR protocol. Inter-assay reproducibility was evaluated on a single batch of 8E5 cells run on three separate occasions by three separate technicians. Mean values, within-run %CV, run-to-run %CV and total %CV were derived for each concentration in the dilution series. Six replicates per run were tested at the 3 input cells dilution, with 3 replicates per run at the other dilutions.

The precision of the assay was evaluated using the replicates from the intra- and inter-assay variability experiments. The means, standard deviations and coefficients of variation were calculated for the 30 replicates run at 3 copies, and 15 replicates run at higher dilutions.

#### Assay specificity for HIV-1

The specificity of the assay for HIV-1 was determined using the standard non-nested RT-qPCR RNA protocol by demonstrating lack of a specific signal in the EDTA plasma from 10 uninfected donors (Biological Specialty Corporation, Colmar, PA, USA) and lack of cross-reactivity with donor or spiked samples infected with other RNA viruses, including Affymetrix-Valiquant HBV and HCV standards (Affymetrix Inc., Santa Clara, CA, USA), cultured EBV, HSV-1, CMV, VZV and Parvovirus from Zeptometrix (Buffalo, NY, USA) spiked into Basematrix diluent (SeraCare, Milford, MA, USA), HIV-2 NIH Reference samples, and a College of American Pathologists (CAP) respiratory panel, ID2-A 2012, containing Influenza, Parainfluenza, RSV, Adenovirus, Human Metapneumovirus and Coronavirus also spiked into Basematrix diluent (SeraCare, Milford, MA, USA).

 As previously mentioned, the specificity of the assay on crude cellular lysates was determined using 74 HIV-1 negative IAVI protocol L donors (26 male and 46 female, and two donors with unknown gender), 12 chronically infected protocol L donors (8 male and 4 female) and 32 treatment-suppressed male HIV-1 positive donors from the London St. Stephens Trust. The IAVI protocol L study was designed for assay characterization and testing of sample collection methods. Volunteers were enrolled from Kigali, Rwanda and the Kenyatta National Hospital and Kangemi Health Centre in Kenya where subtype A is predominant, followed by D, C and G. The London St. Stephens Trust participants were all confirmed as subtype B (Supplementary Table S5). Assay sensitivity, specificity, positive predictive value, negative predictive value and accuracy were determined using the online calculator—https://www.medcalc.org/calc/diagnostic_test.php.

#### HIV-1 cross-subtype specificity

The cross-subtype specificity and accuracy of the assay was determined using well-characterized diversity panels from the U.S. Military’s HIV Research Program (MHRP) and EQAPOL. The MHRP isolates represented an established 60-member international panel consisting of 10 isolates from each of the 6 Major HIV-1 subtypes (https://www.hivreagentprogram.org, Catalog Number ARP 11412). The EQAPOL isolates represented a broad range of recently sourced transmitter/founder (T/F) viral strains of HIV-1 including all major subtypes, common circulating recombinant forms (CRFs) and a few unique recombinant forms (URFs) (EQAPOL, duke.edu). The custom panel included all the transmitted/founder (T/F) viral strains that had been provided to EQAPOL by IAVI and that could be cultured to high titers in primary PBMCs. In total, 127 isolates were tested and comprised of 18 Subtype A, 22 Subtype A recombinants, 18 Subtype B, 17 Subtype C, 2 Subtype C recombinants, 13 Circulating Recombinant F01_AE (CRF01_AE), 12 Subtype D, 2 Subtype F1, 2 Subtype F2, 12 Subtype G, 3 Subtype O and 6 Unique Recombinant Forms (URFs). The EQAPOL-supplied stock samples were diluted 1000-fold in AcroMetrix EDTA plasma dilution matrix (ThermoFisher Scientific) to bring them into the dynamic range of the assay. HIV-1 viral load was expressed in copies per ml, using concentrations determined by the AcroMetrix HIV-1 quantification reference panel (ThermoFisher Scientific).

## Statistical analysis

All data was analyzed using GraphPad Prism 9 (https://www.graphpad.com/scientific-software/pris) and Microsoft Excel unless otherwise stated. A variety of online calculators and tools were utilized wherever they are stated. PCR standard curve statistics were generated by the thermocycler instrument manufacturer’s software packages.

## Supplementary Information

Supplementary Information.

## Data Availability

Detailed raw data files corresponding to Figures 2 and 3; Tables 1,3,4 and 5 and Supplementary Table 2, as well as other data mentioned but not shown in the results section, have been deposited in a "center for open science" database and can be viewed via this link https://osf.io/m9yne/?view_only=47cbcfd3d39b4a89a0d758a474441e38.
