# Author Correction: Development of a sensitive, quantitative assay with broad subtype specificity for detection of total HIV-1 nucleic acids in plasma and PBMC

**DOI:** 10.1038/s41598-022-06286-5

**Published:** 2022-02-01

**Authors:** C. N. Kibirige, M. Manak, D. King, B. Abel, H. Hack, D. Wooding, Y. Liu, N. Fernandez, J. Dalel, Steve Kaye, N. Imami, L. Jagodzinski, J. Gilmour

**Affiliations:** 1 IAVI, Human Immunology Laboratory, Imperial College London, Chelsea and Westminster NHS Foundation Trust, 369 Fulham Road, London, SW10 9NH UK; 2 Henry M. Jackson Foundation for the Advancement of Military Medicine, Bethesda, MD USA; 3 U.S. Military HIV Research Program, Walter Reed Army Institute of Research, 503 Robert Grant Ave., Silver Spring, MD 20910 USA; 4 Diagnostics and Countermeasures Branch, Walter Reed Army Institute of Research, 503 Robert Grant Ave, Silver Spring, MD 20910 USA; 5 Molecular Diagnostics Unit, Imperial College London, Jefferiss Trust Laboratory, St. Mary’s Campus, Norfolk Place, London, W2 1PG UK; 6 Centre for Immunology and Vaccinology, Imperial College London, Chelsea and Westminster NHS Foundation Trust, 369 Fulham Road, London, SW10 9NH UK; 7 Present Address: Turesol Consulting, 314 S. Henderson Road, King of Prussia, PA 19406 USA

Correction to: *Scientific Reports* https://doi.org/10.1038/s41598-021-03016-1, published online 28 January 2022

The original version of this Article contained errors.

In the Results section, under the subheading ‘Assay specificity for HIV‑1’,

“They comprised of 27 male and 47 female participants.”

now reads:

“They comprised of 26 male and 46 female participants and 2 donors with unknown gender.”

In the Methods section, under the subheading ‘Assay specificity for HIV‑1’,

“As previously mentioned, the specificity of the assay on crude cellular lysates was determined using 74 HIV-1 negative IAVI protocol L donors (27 male and 47 female), 12 chronically infected protocol L donors (8 male and 4 female) and 32 treatment-suppressed male HIV-1 positive donors from the London St. Stephens Trust.”

now reads:

“As previously mentioned, the specificity of the assay on crude cellular lysates was determined using 74 HIV-1 negative IAVI protocol L donors (26 male and 46 female, and two donors with unknown gender), 12 chronically infected protocol L donors (8 male and 4 female) and 32 treatment-suppressed male HIV-1 positive donors from the London St. Stephens Trust.”

The original Article has been corrected.

