# Author Correction: Development of a sensitive, quantitative assay with broad subtype specificity for detection of total HIV-1 nucleic acids in plasma and PBMC

**DOI:** 10.1038/s41598-022-16308-x

**Published:** 2022-07-11

**Authors:** C. N. Kibirige, M. Manak, D. King, B. Abel, H. Hack, D. Wooding, Y. Liu, N. Fernandez, J. Dalel, Steve Kaye, N. Imami, L. Jagodzinski, J. Gilmour

**Affiliations:** 1 IAVI, Human Immunology Laboratory, Imperial College London, Chelsea and Westminster NHS Foundation Trust, 369 Fulham Road, London, SW10 9NH UK; 2 Henry M. Jackson Foundation for the Advancement of Military Medicine, Bethesda, MD USA; 3 U.S. Military HIV Research Program, Walter Reed Army Institute of Research, 503 Robert Grant Ave, Silver Spring, MD 20910 USA; 4 Diagnostics and Countermeasures Branch, Walter Reed Army Institute of Research, 503 Robert Grant Ave, Silver Spring, MD 20910 USA; 5 Molecular Diagnostics Unit, Imperial College London, Jeferiss Trust Laboratory, St. Mary’s Campus, Norfolk Place, London, W2 1PG UK; 6 Centre for Immunology and Vaccinology, Imperial College London, Chelsea and Westminster NHS Foundation Trust, 369 Fulham Road, London, SW10 9NH UK; 7 Present Address: Turesol Consulting, 314 S. Henderson Road, King of Prussia, PA 19406 USA

Correction to: *Scientific Reports* 10.1038/s41598-021-03016-1, published online 28 January 2022

The original version of this Article contained errors in the HPRT Reverse Primer and Probe primer sequences, which should read as: Antisense (HPRT-R) GGTCCTTTTCACCAGCAAGCT and Probe (HPRT-P) CTTTCCTTGGTCAGGCAGTATAATC. As a result, in the Materials and methods section, under the subheading ‘Semi-nested RTqPCR for sample enrichment’,

“The qPCRBIO 1-Step Go Lo-Rox Kit reagents (PCR Biosystems) and manufacturer recommended cycling parameters were used with 900 nM TGACACTGGCAAAACAATGCA HPRT Forward Primer, 900 nM AGCTTGCTGGTGAAAAGGACC HPRT Reverse Primer and 250 nM TTTCCTTGGTCAGGCAGTATAATC VIC/TAMRA Probe (ThermoFisher Scientific).”

now reads:

“The qPCRBIO 1-Step Go Lo-Rox Kit reagents (PCR Biosystems) and manufacturer recommended cycling parameters were used with 900 nM TGACACTGGCAAAACAATGCA HPRT Forward Primer, 900 nM GGTCCTTTTCACCAGCAAGCT HPRT Reverse Primer and 250 nM CTTCCTTGGTCAGGCAGTATAATC VIC/TAMRA Probe (ThermoFisher Scientific).”

Additionally, there was an error in 574P and 599R sequences in Table 2, where “I” should read “N”. Thermofisher does not provide the oigonucletides with an Inosine base. They provide equimolar amounts of each possible base in this position.

**Table:** 

574P	LTR 574 ← 552	Probe (total RNA or DNA)	5′-FAM/ACAGA**Y**GGGCACACAC**I**ACT/MGBNFQ-3′
599R	LTR 599 ← 582	Reverse primer (total RNA or DNA)	5′-AGGGATCTCTAG**I**TACCA-3′

should read:

**Table:** 

574P	LTR 574 ← 552	Probe (total RNA or DNA)	5′-FAM/ACAGAYGGGCACACAC**N**ACT/MGBNFQ-3′
599R	LTR 599 ← 582	Reverse primer (total RNA or DNA)	5′-AGGGATCTCTAG**N**TACCA-3′

The original Article has been corrected.

