# Supplementary material for: Development of a sensitive, quantitative assay with broad subtype specificity for detection of total HIV-1 nucleic acids in plasma and PBMC
Source: Sci Rep. 2022 Jan 28;12:1550. doi: 10.1038/s41598-021-03016-1 (PMC8799642; doi:10.1038/s41598-021-03016-1)
Supplement: Supplementary file 1 — Supplementary Information. [file 41598_2021_3016_MOESM1_ESM.pdf]

Title:

Development of a Sensitive, Quantitative Assay with Broad Subtype Specificity for Detection of Total HIV-1 Nucleic Acids in Plasma and PBMC.

Authors:

*Kibirige C. N[1]., Manak M[2,3,4]., King D[1]., Abel B[1]., Hack H[2,3,5]., Wooding D[1]., Liu Y[2,3,5]., Fernandez N[1]., Dalel J[1]., Steve Kaye[6], Imami N[7], Jagodzinski L[3,5]., Gilmour J[1].

Author Affiliations:

[1]IAVI, Human Immunology Laboratory, Imperial College London, Chelsea & Westminster NHS Foundation Trust, 369 Fulham Road, London, SW10 9NH, UK.
[2] Henry M. Jackson Foundation for the Advancement of Military Medicine, Bethesda, MD, USA.
[3]U.S. Military HIV Research Program, Walter Reed Army Institute of Research, 503 Robert Grant Ave., Silver Spring, MD 20910, USA.
[4]Current Affiliation – Turesol Consulting, 314 S. Henderson Road, King of Prussia PA, 19406, USA.
[5]Diagnostics and Countermeasures Branch, Walter Reed Army Institute of Research, 503 Robert Grant Ave, Silver Spring, MD 20910, USA.
[6]Molecular Diagnostics Unit, Imperial College London, Jefferiss Trust Laboratory, St. Mary's Campus, Norfolk Place, London, W2 1PG
[7]Centre for Immunology and Vaccinology, Imperial College London, Chelsea & Westminster NHS Foundation Trust, 369 Fulham Road, London, SW10 9NH, UK.

Corresponding Author Address:

*c.kibirige@imperial.ac.uk.

Supplementary Figures and Tables

Supplementary Table 1. Oligonucleotides initially evaluated for sensitivity, linearity, precision and subtype specificity.
All primers and probes were synthesized at Integrated DNA Technologies, IA, USA (IDT). [a] Genbank Accession ID K03455. [b] These oligonucleotides were selected for further optimization after favorable initial performance on an ACH-2 cell lysate dilution series using universal cycling conditions. The ACH-2 cell line is a standard that was engineered to contain 1 copy of integrated HIV-1 DNA per cell (https://www.hivreagentprogram.org/ Cat No. ARP-349).
[c] The original assay containing the 546P oligonucleotide used two probes in a "Molecular Beacon" format. In our protocol, 546P was used as a single TaqMan probe containing FAM Dye and ZEN/Iowa Black Double Quencher modifications.

Oligo Name	HXB2[a] nt Position	Original Citation	Sequence
[b]496F	LTR 496→516	Brussel et al. 2005	5'-GGCTAACTAGGGAACCCACTG-3'
523F	LTR 523→544	MacNeil et al. 2006	5'-CCTCAATAAAGCTTGCCTTGAG-3'
525F	LTR 525→542	Friedrich et al. 2010	5'-GCCTCAATAAAGCTTGCCTTGA-3'
[bc]546P	LTR 522←546	Brussel et al. 2005	5'-/56-FAM/CACTCAAGG/ZEN/CAAGCTTTATTGAGGC/3IABkFQ/-3'
556P	LTR 556→582	Friedrich et al. 2010	5'-CACAACAGACGGGCACACACTTGA-3'
557F	LTR 557→576	Butler et al. 2001	5′-TGTGTGCCCGTCTGTGT-3'
572P	LTR 572→549	Brussel et al. 2005	5'-CACAACAGACGGGCACACACTTGA-3'
[b]622R	LTR 599←622	Friedrich et al. 2010	5'-TCCACACTGACTAAAAGGGTCTGA–3'
633P	GAG 652←633	Butler et al. 2001	5'–CAGTCGCGCCCGAACAGGGA–3'
[b]633R	LTR 633→609	Brussel et al. 2005	5'-GCTAGAGATTTTCCACACTGACTAA -3'
[b]λ496F	LTR 496→516	Brussel et al. 2005	5'-ATGCCACGTAAGCGAAACTGGCTAACTAGGGAACCCACTG-3'
699R	GAG 680←699	Butler et al. 2001	5' -GAGTCCTGCGTCGAGAGAGC-3'
[b]λT	Lambda Tag	Brussel et al. 2005	5′ -ATGCCACGTAAGCGAAACT-3′

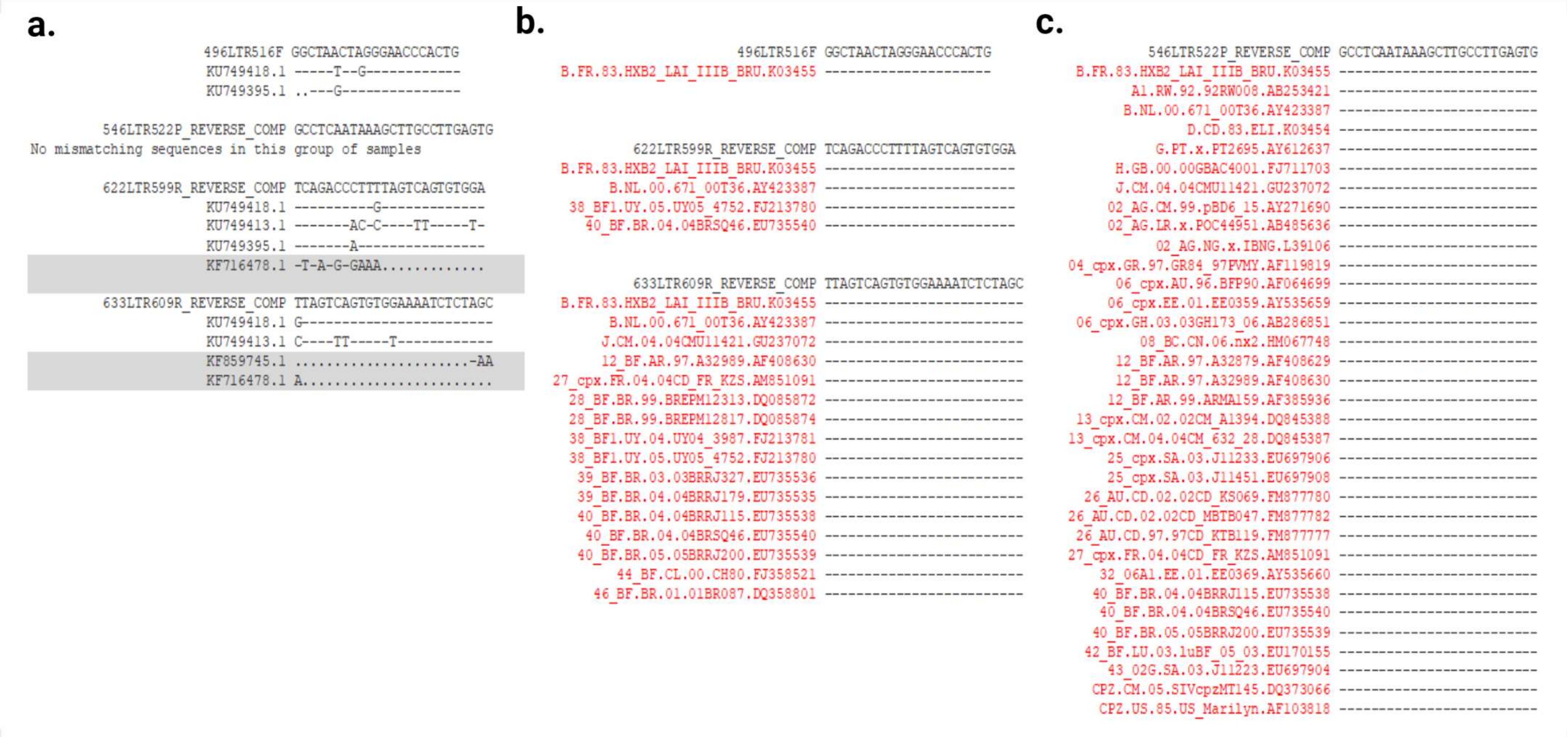

Supplementary Figure 1. a) Sequence alignment of Brussel/Friedrich (496F/546P/622R1/633R2) oligonucleotides against published sequences of the EQAPOL sub-panel of 20 HIV-1 variants showing only accession numbers with mismatches; Grey shading indicates sample ID that was successfully detected by the assay despite the indicated mismatches. b) Alignment of 496F, 622R1 & 633R2 oligonucleotides against NCBI/LANL 2005 HIV compendium sequences showing only perfect matches; c) Alignment of 546P oligonuceotide against the NCBI/LANL 2005 HIV compendium sequences showing only perfect matches (https://www.hiv.lanl.gov/content/sequence/HIV/COMPENDIUM/2005/RefSeqs05.pdf, www.hiv.lanl.gov).

Supplementary Table 2. Summary of results from the alignment of the primer probe set used in the current assay as compared to those of the Brussel 2005(Freidrich 2010 internal Reverse Primer), Vandergeeten 2014, van der Sluis 2013, Schvachsa 2007 or Viard 2004 oligonucleotides against the HIV compendium database.

	N	% Mismatch		N	% Mismatch		N	% Mismatch		N	% Mismatch	
		0-1	>1		0-1	>1		0-1	>1		0-1	>1
Current assay	525F			574P			599R					
Aligned Seqs	64	77	23	83	70	30	85	95	5			
Brussel 2005 *(Freidrich 2010 Internal Reverse Primer)*	496F			546P			622R			633R		
Aligned Seqs	53	45	55	70	60	40	89	17	83	101	26	74
Vandergeeten 2014	452F			522P			775R			583R		
Aligned Seqs	45	64	36	64	63	37	179	72	28	85	91	8
van der Sluis 2013	522F			584P			643R					
Aligned Seqs	64	40	24	86	94	6	114	71	29			
Schvachsa 2007	522F			551P			642R					
Aligned Seqs	63	67	33	79	67	33	114	75	25			
Viard 2004	522F			551P			642R					
Aligned Seqs	64	66	34	84	54	46	114	76	24			

N = number of sequences examined including at least one (up to 6) isolates from each of subtype A, B, C, D, CRFO1_AE, F, CRF02_AG, and G.

The % of isolates that have one or no mismatches or more than 1 mismatch in the indicated primer is shown.

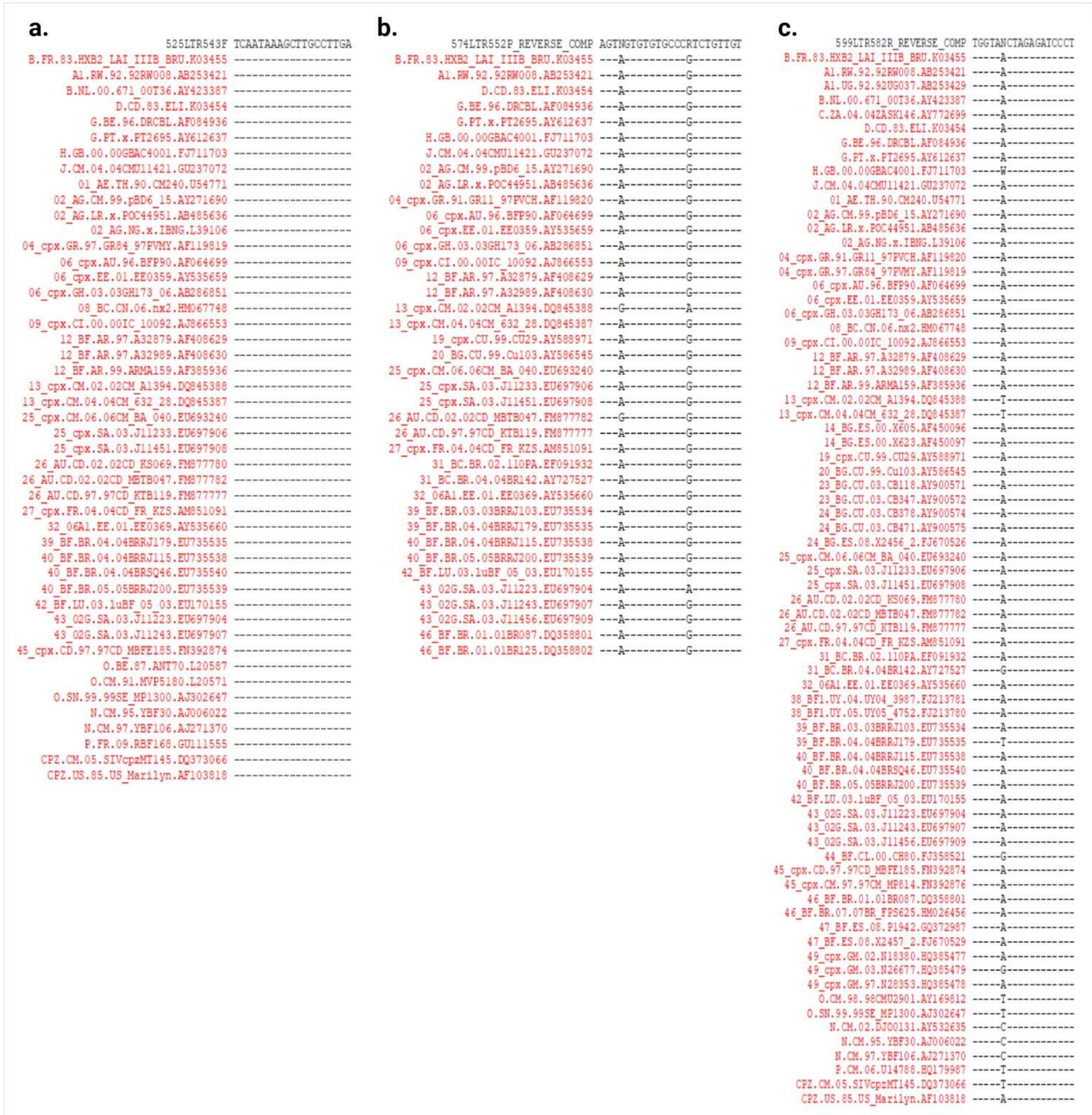

Supplementary Figure 2.
Sequence alignment of a) 525F b) 574P and c) 599R assay oligonucleotides from the revised assay against the NCBI/LANL 2005 HIV compendium sequences showing only perfect matches (https://www.hiv.lanl.gov/content/sequence/HIV/COMPENDIUM/2005/RefSeqs05.pdf, www.hiv.lanl.gov

Supplementary Table 3. Performance of the assay (525F/574P/599R) in a semi-nested RT-qPCR format on an extended diversity panel of spiked plasmas incorporating all the transmitted founder viruses (TFVs) submitted by IAVI to the External Quality Assurance Program Oversight Laboratory (EQAPOL). Viral loads were determined by EQAPOL using the Roche Cobas AmpliPrep/Cobas TaqMan HIV-1 test v2.0 assay (Roche Diagnostics) and compared to values obtained using the assay. Variants that were tested in the initial sub-panel of 20 variants are shown in bold-type.

[1] *Brown et al 2005. NIH AIDS Reagent program (www.aidsreagent.org) Cat No. 11413 International Panel.*
[2] *Year of sampling is not publicly available. Year of GENBANK submission reported instead*

GenBank/ Sample ID	Subtype	Country of Origin	Year of Sampling / Submission[2]	Roche COBAS®	LDA	Isolate Source
**KF859745**	**A1**	**Uganda**	**2010**	**1.43e+04**	**5.98e+04**	**EQAPOL**
**KF716472**	**A1**	**Rwanda**	**2011**	**6.63e+04**	**5.49e+05**	**IAVI/EQAPOL**
**KF716478**	**A1**	**Uganda**	**2009**	**1.13e+05**	**1.46e+06**	**IAVI/EQAPOL**
KP109490	A1	Uganda	2009	4.29e+04	2.50e+06	EQAPOL
KF716475	A1	Kenya	2011	3.81e+04	4.83e+05	IAVI/EQAPOL
KF716474	A1	Kenya	2013	4.24e+04	9.08e+05	IAVI/EQAPOL
KU749423	A1	Rwanda	2006	4.46e+04	1.44e+06	IAVI/EQAPOL
KU749424	A1	Rwanda	2008	5.88e+04	1.22e+06	IAVI/EQAPOL
KU749429	A1, A2, C	Kenya	2007	6.81e+04	6.95e+05	IAVI/EQAPOL
**KU749427**	**A1, A2, D**	**Kenya**	**2006**	**6.07e+04**	**2.90e+05**	**IAVI/EQAPOL**
KF716469	A1, C	Kenya	2010	4.50e+04	8.45e+05	IAVI/EQAPOL
KU749431	A1, C	Kenya	2011	4.90e+04	1.30e+06	IAVI/EQAPOL
KU749428	A1, C	Rwanda	2006	3.66e+04	2.71e+05	IAVI/EQAPOL
KF716489	A1, C, D	Uganda	2010	8.59e+04	1.27e+06	EQAPOL
KF716470	A1, C, D	Kenya	2010	2.55e+04	8.63e+05	IAVI/EQAPOL
KU749430	A1, C, D	Kenya	2008	4.19e+04	1.86e+05	IAVI/EQAPOL
KF716482	A1, D	Uganda	2010	5.38e+04	3.51e+05	IAVI/EQAPOL
KF716468	A1, D	Kenya	2009	4.33e+04	1.22e+06	IAVI/EQAPOL
KF716487	A1, D	Uganda	2010	2.48e+04	4.60e+05	IAVI/EQAPOL
KF716485	A1, D	Uganda	2011	3.55e+04	1.07e+05	EQAPOL
KF716483	A1, D	Uganda	2010	6.62e+04	3.79e+06	EQAPOL
KF859746	A1, D	Uganda	2009	5.87e+04	1.77e+05	IAVI/EQAPOL
KF716484	A1, D	Uganda	2011	2.85e+04	1.03e+06	IAVI/EQAPOL
KF716490	A1, D	Uganda	2010	2.63e+04	2.62e+05	IAVI/EQAPOL
KF859747	A1, D	Uganda	2009	4.92e+04	3.13e+05	EQAPOL
KU749421	A1, F2, G	Cameroon	2010	4.22e+04	1.97e+06	EQAPOL
**KU749414**	**A1, G**	**Pakistan**	**2014**	**1.21e+05**	**4.14e+05**	**EQAPOL**
**KU749413**	**A1, G**	**Pakistan**	**2014**	**1.02e+05**	**3.25e+05**	**EQAPOL**
**KF716488**	**A1, G, CRF01_AE**	**Uganda**	**2010**	**5.04e+04**	**2.89e+05**	**IAVI/EQAPOL**
**KU749431**	**A1, C**	**Kenya**	**2011**	**4.90e+04**	**1.29e+06**	**IAVI/EQAPOL**
AY713407	A	Uganda	[2]1992	1.63e+05	1.22e+05	[1]MHRP/NIH
AY713406	A	Rwanda	[2]2004	1.74e+05	7.64e+04	[1]MHRP/NIH
AF457052	A	Kenya	[2]2001	2.09e+05	3.12e+04	[1]MHRP/NIH
AF457057	A	Kenya	[2]2001	1.22e+05	2.40e+04	[1]MHRP/NIH
AF457063	A	Kenya	[2]2001	1.08e+05	1.05e+05	[1]MHRP/NIH
AF457065	A	Kenya	[2]2002	1.54e+05	9.05e+04	[1]MHRP/NIH

AF457066	A	Kenya	[2]2002	1.70e+05	9.26e+04	[1]MHRP/NIH
AF457068	A	Kenya	[2]2001	1.76e+05	1.55e+05	[1]MHRP/NIH
AF457069	A	Kenya	[2]2001	3.11e+05	2.78e+05	[1]MHRP/NIH
AF457079	A	Kenya	[2]2001	2.80e+05	1.61e+05	[1]MHRP/NIH
**KC473835**	**B**	**USA**	**2011**	**3.50e+04**	**8.51e+04**	**EQAPOL**
KC473833	B	USA	2011	4.36e+04	4.36e+05	EQAPOL
KC473832	B	USA	2011	6.87e+04	3.39e+05	EQAPOL
KF716497	B	Japan	2011	5.61e+04	1.65e+06	EQAPOL
KU749387	B	USA	2013	3.68e+04	5.79e+05	EQAPOL
KF716496	B	France	2011	6.16e+04	5.27e+05	EQAPOL
KC473831	B	USA	2011	7.04e+04	1.93e+05	EQAPOL
KC473834	B	USA	2011	6.85e+04	4.83e+05	EQAPOL
M17449	B	USA	1984	1.89e +05	2.90e+05	[1]MHRP/NIH
AY713409	B	USA	[2]2004	2.61e +05	2.97e+05	[1]MHRP/NIH
AY173952	B	USA	[2]2002	2.21e +05	7.74e+04	[1]MHRP/NIH
AY173955	B	USA	[2]2002	2.06e+05	3.14e+05	[1]MHRP/NIH
AY173951	B	Thailand	[2]2002	2.13e+05	3.16e+05	[1]MHRP/NIH
AY173956	B	Brazil	[2]2002	2.44e+05	1.39e+05	[1]MHRP/NIH
AY713410	B	USA	[2]2004	1.31e+05	2.08e+05	[1]MHRP/NIH
AY713412	B	USA	[2]2004	1.97e+05	1.90e+05	[1]MHRP/NIH
AY713408	B	Thailand	[2]2004	1.79e+05	2.38e+05	[1]MHRP/NIH
AY713411	B	France	[2]2004	1.45e+05	8.06e+04	[1]MHRP/NIH
**KF716467**	**C**	**Zambia**	**2011**	**5.25e+04**	**9.81e+04**	**IAVI/EQAPOL**
**KU749425**	**C**	**Zambia**	**2007**	**5.27e+04**	**2.98e+05**	**IAVI/EQAPOL**
KU749426	C	Zambia	2009	4.26e+04	1.61e+06	IAVI/EQAPOL
KP109494	C	Zambia	2011	7.38e+04	2.65e+05	IAVI/EQAPOL
KP109495	C	Zambia	2011	4.61e+04	1.94e+05	IAVI/EQAPOL
KP109496	C	Zambia	2011	5.56e+04	3.45e+05	IAVI/EQAPOL
KF716466	C	Zambia	2009	5.87e+04	6.74e+05	IAVI/EQAPOL
KF716473	C, D	Rwanda	2011	6.08e+04	2.28e+05	IAVI/EQAPOL
**KU749418**	**C, U**	**South Africa**	**2013**	**4.08e+04**	**1.54e+04**	**IAVI/EQAPOL**
AY713413	C	Malawi	[2]2004	1.22e+05	9.32e+04	[1]MHRP/NIH
AY713414	C	India	[2]2004	3.05e+04	*0.00e+00	[1]MHRP/NIH
AY713416	C	Senegal	[2]2004	8.99e+04	4.87e+04	[1]MHRP/NIH
AY713415	C	Somalia	[2]2004	1.30e+05	7.54e+04	[1]MHRP/NIH
AY444801	C	USA	[2]2003	1.15e+05	9.97e+04	[1]MHRP/NIH
AY255825	C	Ethiopia	[2]2003	1.94e+05	1.89e+05	[1]MHRP/NIH
AY253304	C	Tanzania	[2]2003	2.06e+05	2.28e+05	[1]MHRP/NIH
AY253308	C	Tanzania	[2]2003	1.21e+05	9.21e+04	[1]MHRP/NIH
AY253322	C	Tanzania	[2]2003	1.97e+05	1.17e+05	[1]MHRP/NIH
AY713417	C	Ethiopia	[2]2004	4.22e+05	4.74e+05	[1]MHRP/NIH
**KC596065**	**CRF01_AE**	**China**	**2011**	**6.58e+04**	***0.00e+00**	**EQAPOL**
KC596064	CRF01_AE	China	2011	5.74e+04	1.34e+06	EQAPOL
KC596063	CRF01_AE	China	2010	6.87e+04	6.42e+05	EQAPOL
AF259954, AF259955	CRF01_AE	Thailand	[2]2000	2.24e+06	1.34e+06	[1]MHRP/NIH
U54771	CRF01_AE	Thailand	[2]1996	7.16e+05	2.12e+05	[1]MHRP/NIH

AY713425	CRF01_AE	Thailand	[2]2004	5.51e+04	1.55e+06	[1]MHRP/NIH
AY713424	CRF01_AE	Thailand	[2]2004	1.24e+05	4.92e+04	[1]MHRP/NIH
AY713423	CRF01_AE	Thailand	[2]2004	6.98e+05	3.48e+04	[1]MHRP/NIH
AY713422	CRF01_AE	Thailand	[2]2004	5.24e+05	3.62e+05	[1]MHRP/NIH
AY713426	CRF01_AE	Thailand	[2]2004	4.26e+05	3.92e+05	[1]MHRP/NIH
AY713421	CRF01_AE	Thailand	[2]2004	1.35e+05	8.35e+04	[1]MHRP/NIH
AY713420	CRF01_AE	Thailand	[2]2004	5.56e+04	1.69e+04	[1]MHRP/NIH
AY713419	CRF01_AE	Thailand	[2]2004	1.71e+05	1.20e+05	[1]MHRP/NIH
**KF716480**	**D**	**Uganda**	**2011**	**1.40e+05**	**1.92e+04**	**IAVI/EQAPOL**
KF716479	D	Uganda	2010	4.75e+04	8.87e+10	IAVI/EQAPOL
AY713418	D	Uganda	[2]2004	1.14e+05	1.53e+05	[1]MHRP/NIH
AF484502	D	Uganda	[2]2002	1.36e+05	2.76e+05	[1]MHRP/NIH
AF484518	D	Uganda	[2]2002	1.07e+05	7.75e+04	[1]MHRP/NIH
AF484477	D	Uganda	[2]2002	1.34e+05	2.30e+05	[1]MHRP/NIH
AY304496	D	Uganda	[2]2003	1.23e+05	1.66e+05	[1]MHRP/NIH
AF484516	D	Uganda	[2]2002	1.61e+05	9.36e+04	[1]MHRP/NIH
AF484486	D	Uganda	[2]2002	6.62e+04	8.20e+04	[1]MHRP/NIH
AF484487	D	Uganda	[2]2002	1.30e+05	8.27e+04	[1]MHRP/NIH
AF484515	D	Uganda	[2]2002	1.30e+05	1.02e+05	[1]MHRP/NIH
AF457090	D	Kenya	[2]2001	1.79e+05	1.26e+04	[1]MHRP/NIH
**JX140671**	**F1**	**Spain**	**2010**	**2.80e+04**	**2.86e+04**	**EQAPOL**
**KU749395**	**F1**	**Brazil**	**2010**	**4.31e+04**	**1.08e+05**	**EQAPOL**
**JX140673**	**F2**	**Cameroon**	**2010**	**7.16e+04**	**1.53e+05**	**EQAPOL**
JX140672	F2	Cameroon	2010	1.25e+05	1.20e+06	EQAPOL
**JX140676**	**G**	**Cameroon**	**2010**	**1.53e+05**	**1.17e+05**	**EQAPOL**
KF716477	G	Kenya	2009	4.54e+04	1.24e+05	IAVI/EQAPOL
AF063223	G	Djibouti	[2]1998	9.28e+04	6.08e+04	[1]MHRP/NIH
AY444808	G	USA	[2]2003	1.42e+05	1.44e+05	[1]MHRP/NIH
AY371122	G	Cameroon	[2]2003	1.24e+05	7.75e+04	[1]MHRP/NIH
AY371123	G	Cameroon	[2]2003	1.32e+05	2.31e+04	[1]MHRP/NIH
AY371124	G	Cameroon	[2]2003	1.51e+05	2.14e+05	[1]MHRP/NIH
AY371125	G	Cameroon	[2]2003	1.68e+05	3.55e+05	[1]MHRP/NIH
AY371126	G	Cameroon	[2]2003	1.06e+05	2.00e+05	[1]MHRP/NIH
AY371127	G	Cameroon	[2]2003	1.73e+05	1.25e+05	[1]MHRP/NIH
AY371138	G	Cameroon	[2]2003	1.64e+05	2.16e+05	[1]MHRP/NIH
AF063223	G	Djibouti	[2]1998	1.42e+05	1.44e+05	[1]MHRP/NIH
**KF859742**	**O**	**Germany**	**2012**	**5.42e+04**	**4.45e+06**	**EQAPOL**
KF859743	O	Spain	2012	1.45e+05	9.66e+05	EQAPOL
KF859744	O	USA	2012	5.39e+04	6.45e+05	EQAPOL
**KP109492**	**URF_A1D**	**Uganda**	**2009**	**2.81e+04**	**6.11e+04**	**IAVI/EQAPOL**
KP109497	URF_A1D	Uganda	2011	4.78e+04	3.90e+06	IAVI/EQAPOL
KP109491	URF_A1D	Uganda	2009	4.49e+04	1.52e+06	IAVI/EQAPOL
KP174771	URF_A1D	Kenya	2009	3.12e+04	2.08e+05	IAVI/EQAPOL
KP109493	URF_A1D	Uganda	2010	4.80e+04	1.29e+06	IAVI/EQAPOL
KF16488	URF_01A1G	Uganda	2010	1.43e+04	5.98e+04	IAVI/EQAPOL

Supplementary Table 4. Revised Assay Formats, Recommended Use and Limitations

Assay Format	Recommended Uses and Other Utilities	Oligonucleotides, Cycling Parameters and Recommended Reagents		Limitations and Cautions
		1st Round	2nd Round	
Non-nested qPCR	• Simplest and most cost-effective assay format<br>• Recommended for quantifying cell-associated DNA even from crude lysates<br>• Can be used for DNA from other sources.<br>• Detects cell-associated HIV-1 even when it is non-detectable in plasma – i.e., highly sensitive.<br>• Recommended for resource-limited settings.<br>• Can be used as a proxy for the Quantitative Viral Outgrowth Assay (QVOA) in cure research studies	• 525F/574P/599R.<br>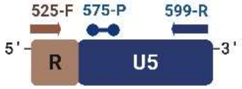<br>• Step-Up Cycling<br>• High-sensitivity master mix that accommodates PCR inhibitors in crude lysates e.g., PCRBIO Probe Mix	• n/a	• For use with DNA Only.<br>• Crude cellular lysates are prone to false positive PCR results.<br>• Does not distinguish between intact and defective integrated or episomal DNA like the IPDA.
Non-nested RTqPCR	• Recommended for cell-associated and fluid-based RNA including Viral Load testing.	• 525F/574P/599R.<br>• Step-up cycling and 20μl reaction volumes for high copy samples<br>• Touch-Up Cycling and 50μl reaction volumes for targets of less than 100 copies/ml<br>• High-sensitivity master mixes e.g., PCRBIO 1-Step Go Mix	• n/a	• 50μl volume required for samples with less than 100 copies/ml.<br>• Sensitivity down to 88 viral RNA copies/ml with 95% confidence.
Semi-nested RTqPCR	• Recommended for cell-associated and fluid-based RNA including Viral Load testing.<br>• Recommended for the target enrichment of very limited or low target-copy samples.<br>• For Research Use.<br>• Highly sensitive.	• λ525F/599R.<br>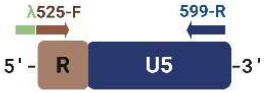<br>• Step-Up Cycling,<br>• 12 cycle pre-amplification<br>• High-sensitivity master mix e.g., PCRBIO 1-Step Go Mix	• λT/574P/599R.<br>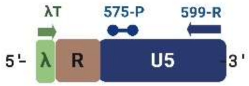<br>• Step-Up Cycling.<br>• Use 2ul of 1st Round product in a 20ul reaction volume (i.e., 10% sample volume).<br>• High sensitivity master mix that is resistant to PCR inhibitors e.g., PCRBIO Probe Mix.	• Highly prone to contamination and false-positives.<br>• Requires a facility specialized for highly sensitive PCR work.<br>• Not recommended for routine clinical use but for specialized research.

Supplementary Table 5. PBMC Specificity Sample Donor Characteristics

NB: IAVI Protocol L samples were derived from Kigali in Rwanda and Kenyatta National Hospital and Kangemi Health Centre in Kenya where subtype A is predominant, followed by D, C and G

All the London St. Stephen's Trust donors were confirmed as harboring HIV-1 subtype B.

Patient Group	Male	Female	Total
IAVI Protocol L,<br>HIV-1 Negative	26	46	74<br>*Including 2 with unknown gender
IAVI Protocol L,<br>HIV-1 Chronically Infected	8	4	12
London St. Stephen's Trust,<br>HIV-1 Positive cART Suppressed	32	0	32
